# Aspartyl proteases in *Candida glabrata* are required for suppression of the host innate immune response

**DOI:** 10.1074/jbc.M117.813741

**Published:** 2018-02-28

**Authors:** Mubashshir Rasheed, Anamika Battu, Rupinder Kaur

**Affiliations:** From the ‡ Centre for DNA Fingerprinting and Diagnostics, Uppal, Hyderabad 500039 and; § Graduate Studies, Manipal Academy of Higher Education, Manipal, Karnataka 576104, India

**Keywords:** cell wall, chemotaxis, interleukin 1 (IL-1), macrophage, spleen tyrosine kinase (Syk), Yapsins, cell wall remodeling, mouse organ colonization, IL-1beta, Syk, THP-1 macrophages, intracellular survival

## Abstract

A family of 11 cell surface-associated aspartyl proteases (CgYps1–11), also referred as yapsins, is a key virulence factor in the pathogenic yeast *Candida glabrata*. However, the mechanism by which CgYapsins modulate immune response and facilitate survival in the mammalian host remains to be identified. Here, using RNA-Seq analysis, we report that genes involved in cell wall metabolism are differentially regulated in the *Cgyps1–11*Δ mutant. Consistently, the mutant contained lower β-glucan and mannan levels and exhibited increased chitin content in the cell wall. As cell wall components are known to regulate the innate immune response, we next determined the macrophage transcriptional response to *C. glabrata* infection and observed differential expression of genes implicated in inflammation, chemotaxis, ion transport, and the tumor necrosis factor signaling cascade. Importantly, the *Cgyps1–11*Δ mutant evoked a different immune response, resulting in an enhanced release of the pro-inflammatory cytokine IL-1β in THP-1 macrophages. Further, *Cgyps1–11*Δ–induced IL-1β production adversely affected intracellular proliferation of co-infected WT cells and depended on activation of spleen tyrosine kinase (Syk) signaling in the host cells. Accordingly, the Syk inhibitor R406 augmented intracellular survival of the *Cgyps1–11*Δ mutant. Finally, we demonstrate that *C. glabrata* infection triggers elevated IL-1β production in mouse organs and that the *CgYPS* genes are required for organ colonization and dissemination in the murine model of systemic infection. Altogether, our results uncover the basis for macrophage-mediated killing of *Cgyps1–11*Δ cells and provide the first evidence that aspartyl proteases in *C. glabrata* are required for suppression of IL-1β production in macrophages.

## Introduction

Hospital-acquired invasive mycoses pose an enormous health and economic challenge and account for 10% of all nosocomial bloodstream infections (BSIs)^4^ worldwide (1, 2). *Candida* species, benign residents of mucosal surfaces and the gut in healthy individuals, are the fourth most common bloodstream pathogens (1–3). Among *Candida* spp., *Candida albicans* is the predominant species responsible for 60% of BSIs (1, 2). However, recent global surveillance programs have revealed a substantial shift in the epidemiology of systemic candidiasis to non-*albicans* species (1–3). Prevalence of *C. glabrata*, a haploid budding yeast, varies geographically, ranges from second to fourth, and accounts for up to 30% of *Candida* BSIs (2–5).

Phylogenetically, *C. glabrata* is more closely related to the nonpathogenic yeast *Saccharomyces cerevisiae* than to other pathogenic *Candida* spp. and belongs to the *Saccharomyces* clade (5, 6). Accordingly, the ability of *C. glabrata* to survive in and adapt to multiple host microenvironments is presumed to emerge independently from that of other *Candida* species (6). *C. glabrata* lacks mating and true hyphae formation and induces no mortality in immunocompetent mice in the systemic candidiasis model (5–7). However, it is able to adhere to biotic and abiotic surfaces via a family of cell wall adhesins, possesses a family of 11 glycosylphosphatidylinositol (GPI)-linked aspartyl proteases, and shows high intrinsic resistance to diverse stresses and azole antifungal drugs (5, 7, 8).

Using macrophage culture and murine models, it has previously been demonstrated that *C. glabrata* is able to proliferate in macrophage cells *in vitro* and evade host immune killing *in vivo* (7, 9–11). In macrophages, *C. glabrata* has been shown to interfere with the phagosomal maturation process, cytokine production, and reactive oxygen species generation (9, 10, 12). Induction of autophagy and transcriptional reprogramming of metabolic genes to survive the nutrient-poor macrophage environment and remodeling of its chromatin architecture to encounter DNA damage stress are known strategies that *C. glabrata* employs to replicate in macrophages (12, 13).

Among known virulence factors of *C. glabrata*, a family of 11 putative GPI-linked, cell surface-associated aspartyl proteases occupies a central position (7, 9). These proteases, also referred as yapsins, are encoded by *CgYPS1–11* genes. Of these, eight *CgYPS* genes (*CgYPS3–6* and *CgYPS8–11*) are encoded in a unique cluster on chromosome E (9). *CgYPS* genes show structural similarity to five *YPS* genes (*YPS1–3*, *YPS6*, and *YPS7*) present in *S. cerevisiae* (9, 14). Unlike most aspartyl proteases, which cleave at hydrophobic residues, yapsins have a common specificity for basic amino acid residues (14, 15).

Of the 11 *CgYPS* genes, seven (*CgYPS2*, *CgYPS4–5*, and *CgYPS8–11*) are up-regulated in response to internalization by macrophages (9). Accordingly, CgYapsins have been implicated in survival of *C. glabrata* in macrophages, cell wall remodeling, activation of macrophages through nitric oxide generation, and virulence in both a systemic model of candidiasis and a minihost model of *Drosophila melanogaster* (9, 12, 16, 17). The role of CgYapsins in cell wall homeostasis has been attributed in part to the removal and release of GPI-anchored cell wall proteins (9). In addition, CgYapsins have been implicated in proper functioning of the vacuole (16), with CgYps1 also uniquely required for intracellular pH homeostasis (18).

Because survival of *C. glabrata* in the host largely relies on an immune evasion mechanism (19) and CgYapsins are essential for its virulence (9), we, here, have examined their biological functions via a combined approach of gene disruption, transcriptional, and immunological analyses. Using human THP-1 macrophages, we show that the putative catalytic aspartate residue of CgYps1 is critical for intracellular survival and proliferation of *C. glabrata*. Our data implicate CgYps4, -5, -8, and -10 for the first time in survival of the macrophage internal milieu. Last, we demonstrate that CgYapsins are required for inhibition of Syk signaling–dependent production of the pro-inflammatory cytokine interleukin (IL)-1β in macrophages and for organ colonization and dissemination during systemic infections of mice.

## Results

### RNA-Seq analysis reveals up-regulation of cell wall organization genes in the Cgyps1–11Δ mutant

The *Cgyps1–11*Δ mutant, which lacks all 11 CgYapsins, is known to display pleiotropic defects, including increased susceptibility to cell wall and osmotic stress, perturbed vacuole and pH homeostasis, and attenuated intracellular survival and pathogenesis (9, 16, 18). Hence, to investigate whether lack of CgYapsins affects gene regulation at the transcriptional level, we performed global transcriptome profiling of YPD-grown log-phase WT and *Cgyps1–11*Δ cells using the RNA-Seq approach. We found a total of 124 genes to be differentially expressed (≥1.5-fold change and a false discovery rate–adjusted *p* value of ≤0.05) in the *Cgyps1–11*Δ mutant (Table S1). Of these, 89 and 35 genes were up-regulated and down-regulated, respectively (Table S1). Gene ontology (GO)-Slim Mapper analysis, using the Candida Genome Database (www.candidagenome.org),^5^ revealed genes involved in biological processes of “transport,” “response to stress,” “carbohydrate metabolic process,” and “cell wall organization” to be differentially expressed in the *Cgyps1–11*Δ mutant.

**Figure 1.**
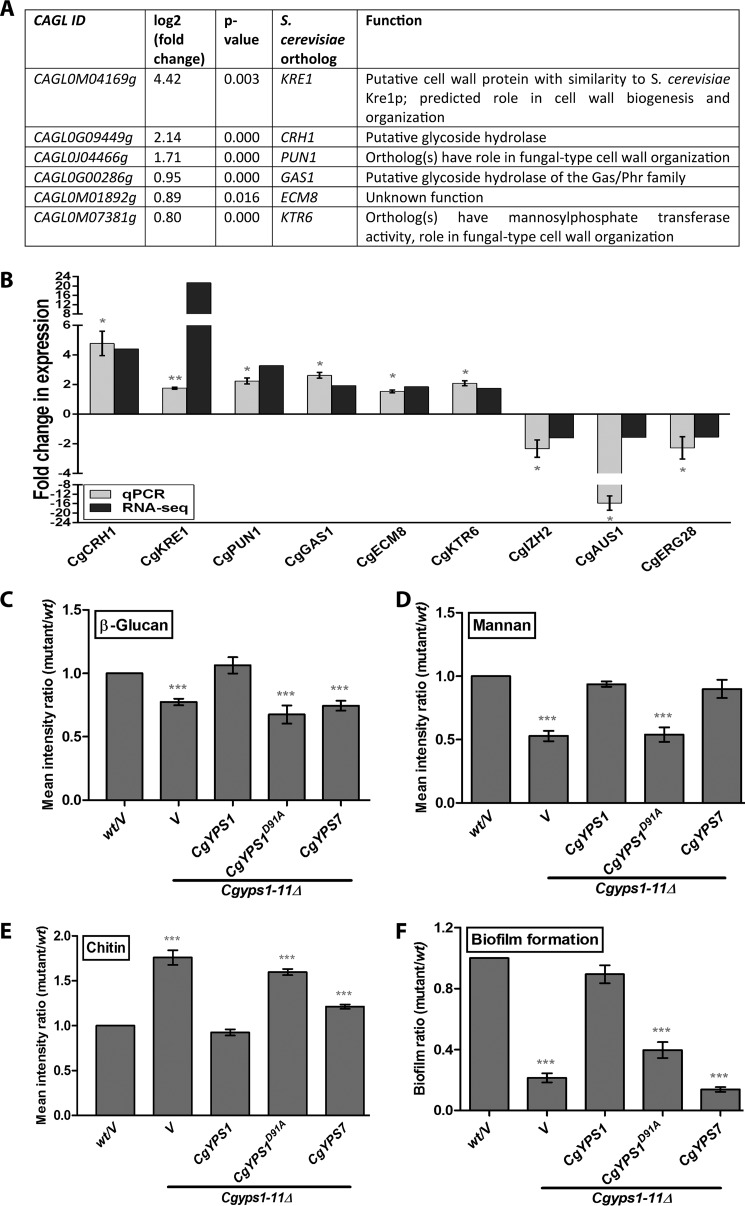
**RNA-Seq analysis reveals genes involved in polysaccharide metabolism to be up-regulated in the *Cgyps1–11*Δ mutant.** *A*, list of differentially expressed genes that belong to the GO term “fungal-type cell wall organization” (GO:0031505), in the *Cgyps1–11*Δ mutant. *B*, qPCR validation of the RNA-Seq data. RNA was extracted, using the acid phenol extraction method, from log-phase WT and *Cgyps1–11*Δ cultures, and transcript levels of the indicated genes (six up-regulated and three down-regulated in the RNA-Seq experiment) were measured by qPCR. Data (mean ± S.E. (*error bars*), *n* = 3–4) were normalized against the *CgACT1* mRNA control and represent -fold change in expression in *Cgyps1–11*Δ mutant compared with the WT strain. *, *p* < 0.05, paired two-tailed Student's *t* test. *C–E*, flow cytometry–based cell wall polysaccharide measurement. Indicated log-phase *C. glabrata* strains were harvested and stained with aniline blue, FITC-concanavalin A, and calcofluor white to estimate cell wall β-glucan (*C*), mannan (*D*), and chitin (*E*) content, respectively. Data (mean ± S.E., *n* = 3–7) presented as the mean fluorescence intensity ratio were calculated by dividing the fluorescence intensity value of the mutant sample by that of the WT sample (set as 1.0). *V*, *C. glabrata* strains carrying empty vector. ***, *p* < 0.001; paired two-tailed Student's *t* test. *F*, assessment of biofilm-forming capacity of the indicated *C. glabrata* strains on polystyrene-coated plates through a crystal violet–based staining assay. YPD-grown log-phase cells were suspended in PBS, and 1 × 10^7^ cells were incubated at 37 °C for 90 min in a polystyrene-coated 24-well plate. After two PBS washes, RPMI medium containing 10% fetal bovine serum was added to each well. Cells were allowed to make biofilms at 37 °C with shaking (75 rpm) for 48 h, with replacement of half of the spent RPMI medium with the fresh medium after 24 h of incubation. Following the removal of unbound *C. glabrata* cells with three PBS washes, the plate was air-dried and incubated with 250 μl of crystal violet solution (0.4% in 20% ethanol). After 45 min, 95% ethanol was added to stained adherent *C. glabrata* cells, and absorbance of the destaining solution was recorded at 595 nm after 45 min. The biofilm ratio was calculated by dividing the mutant absorbance units by those of WT cells (set to 1.0). Data represent mean ± S.E. of 4–7 independent experiments. *V*, *C. glabrata* strains carrying empty vector. ***, *p* < 0.001; paired two-tailed Student's *t* test.

To identify significantly enriched GO terms, classified according to biological process annotations, in differentially expressed genes (DEGs), we employed DAVID (Database for Annotation, Visualization, and Integrated Discovery; https://david.ncifcrf.gov)^5^ (20, 21) and the FungiFun2 tool (https://elbe.hki-jena.de/fungifun/fungifun.php)^5^ (22). GO categories ion transport (GO:0006811; *p* = 0.0002) and oxidation-reduction process (GO:0055114; *p* = 0.0002) were enriched in the down-regulated gene list, and carbohydrate metabolic process (GO:0005975; *p* = 0.0001) was enriched in the up-regulated gene set in the FungiFun2 analysis. GO terms fungal-type cell wall organization (GO:0031505; *p* = 0.0047) and tricarboxylic acid cycle (GO:0006099; *p* = 0.047) were enriched in the up-regulated gene list, and the GO term sterol import (GO:0035376; *p* = 0.0.030) was enriched in the down-regulated gene set in the DAVID analysis. Fungal cell wall organization genes that are differentially expressed in the *Cgyps1–11*Δ mutant are shown in Fig. 1*A*.

We next verified the RNA-Seq gene expression data by quantitative real time-PCR (qPCR) analysis and found a good correlation in expression levels between RNA-Seq and qPCR data for six up-regulated (*CgCRH1*, *CgKRE1*, *CgPUN1*, *CgGAS1*, *CgECM8*, and *CgKTR6*) and three down-regulated (*CgIZH2*, *CgAUS1*, and *CgERG28*) tested genes (Fig. 1*B*). Of note, *CgCRH1*, *CgAUS1*, *CgERG28*, and *CgIZH2* genes code for a putative glycoside hydrolase, sterol transporter, ergosterol biosynthetic protein, and cellular zinc ion homeostatic protein, respectively.

As the *Cgyps1*Δ mutant has previously been shown to display metal ion sensitivity and perturbed pH homeostasis (16, 18), differential expression of genes involved in the oxidation-reduction process and ion transport in the *Cgyps1–11*Δ mutant may be attributed to the role of CgYps1 in maintenance of intracellular pH and ion homeostasis. Further, sunken cell wall and constitutively active protein kinase C–mediated cell wall integrity pathway have previously been reported in the *Cgyps1–11*Δ mutant (16). Thus, our expression profiling data with up-regulation of genes implicated in fungal cell wall organization are consistent with a requirement for CgYapsins for maintenance of the cell wall architecture.

The highly cross-linked fungal cell wall is a dynamic structure that undergoes significant remodeling in response to environmental cues (23). To examine the role of CgYapsins in cell wall homeostasis more closely, we sought to measure levels of cell wall polysaccharides, β-glucan, mannan, and chitin, which account for >90% of the *C. glabrata* cell wall (23), in WT and *Cgyps1–11*Δ cells. Aniline blue–based staining of β-glucan revealed that compared with the WT strain, the *Cgyps1–11*Δ mutant contains 30% lower levels of β-glucan under log-phase conditions (Fig. 1*C*). As *in vitro* phenotypes of *Cgyps1*Δ*7*Δ and *Cgyps1–11*Δ mutants have been reported to be largely similar (9, 16), we next checked CgYps1- and CgYps7-mediated complementation of the *Cgyps1–11*Δ mutant defects. Ectopic expression of *CgYPS1* but not of *CgYPS7* could restore β-glucan content to WT levels (Fig. 1*C*). These results implicate CgYps1 in glucan homeostasis. Similarly, cell wall mannan content, as determined by fluorescence measurement of FITC-labeled concanavalin A-stained cells, was found to be 2-fold lower in the *Cgyps1–11*Δ mutant (Fig. 1*D*). This defect was rescued fully by ectopic expression of either *CgYPS1* or *CgYPS7* (Fig. 1*D*). In contrast, calcofluor white-based staining of cell wall chitin showed 1.8-fold higher chitin content in the *Cgyps1–11*Δ mutant, compared with WT cells (Fig. 1*E*). Importantly, elevated chitin levels in the mutant were fully and partially rescued by expression of *CgYPS1* and *CgYPS7*, respectively (Fig. 1*E*), indicating a role for both CgYps1 and CgYps7 in maintenance of the cell wall chitin content. Together, these data indicate that CgYapsins are required for cell wall homeostasis with a unique requirement of CgYps1 for maintenance of β-glucan content and of CgYps1 and CgYps7 for sustainment of chitin and mannan levels.

Cell wall is the first point of contact between *C. glabrata* cells and the external surface. To investigate whether altered cell wall composition of the *Cgyps1–11*Δ mutant modulates its interaction with abiotic surfaces, we next measured the ability of the mutant to form biofilm on polystyrene-coated microtiter plates. We found that the *Cgyps1–11*Δ mutant was severely compromised in biofilm formation on polystyrene, and *CgYPS1* expression could fully complement this defect (Fig. 1*F*). These results are suggestive of a specific requirement for CgYps1 in biofilm formation and raise the possibility of a nexus between cell wall β-glucan content and the capacity to form biofilm in *C. glabrata*.

Overall, our RNA-Seq and biochemical analysis are demonstrative of an altered cell wall composition of the *Cgyps1–11*Δ mutant, which could largely be attributed to the lack of CgYps1 and CgYps7 enzymes. Furthermore, an explicit function of CgYapsins in cell wall organization is consistent with previous reports (9, 16) as well as with reported roles for yapsins in *S. cerevisiae* (14, 15), indicating functional similarity between aspartyl proteases of these two yeasts.

### Predicted catalytic aspartate residues of CgYps1 are essential for its functions in cell physiology

**Figure 2.**
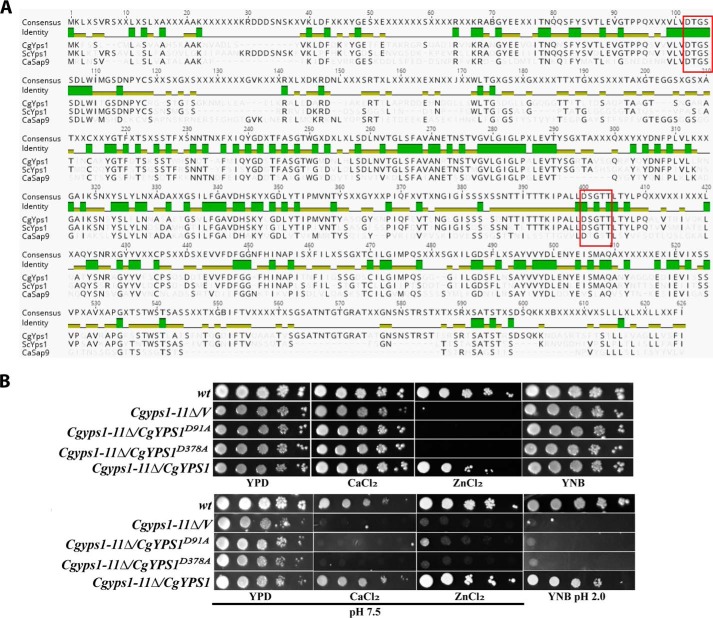
**Predicted catalytic residues of CgYps1 are required for its functions.** *A*, multiple protein sequence alignment of Yps1. The sequences of Yps1 from *C. glabrata* (Cagl0m04191p), *S. cerevisiae* (Ylr120C) and *C. albicans* (CaSap9; C3_03870cp_a) were aligned and colored by the Geneious Basic version 5.6.4 program. Identical and conserved residues are *shaded* in *green* and *greenish-brown colors*, respectively. The conserved catalytic motifs (DTGSS and D(S/T)GTT) are marked with a *red box. B*, serial dilution spotting analysis. Overnight YPD-grown cultures of the indicated strains were taken, and *A*_600_ was normalized to 1.0. 3 μl of 10-fold serially diluted cultures were spotted on YNB, YNB (pH 2.0), and the YPD medium lacking or containing metal ions. Calcium chloride (CaCl_2_) and zinc chloride (ZnCl_2_) were used at a final concentration of 100 and 10 mm, respectively. After growth at 30 °C for 1–3 days, plates were imaged. *V*, *C. glabrata* strains carrying empty vector.

CgYps1 and CgYps7 are putative GPI-anchored cell wall proteases that contain an N-terminal signal peptide, a pro-peptide region, two catalytic domains, a conserved C-terminal region, and a GPI signal at the C terminus (15). These are assumed to undergo proteolytic cleavage with the mature enzyme consisting of α and β subunits, with each subunit contributing one catalytic aspartate residue (15). Although mutants lacking these enzymes displayed cell wall defects (9, 16), the observed defects could presumably be due to the lack of these proteins in the cell wall leading to an altered cell wall structure. Hence, to examine whether presence or the catalytic activity of CgYps1 and CgYps7 is required for cell wall metabolism, we sought to identify and mutate conserved aspartic acid residues in both enzymes. It is worth noting that the *Cgyps7*Δ mutant has previously been shown to be sensitive to cell wall stressors (9). Multiple-protein sequence alignment of *C. glabrata* Yps1, *S. cerevisiae* Yps1, and *C. albicans* Sap9 (CaYps1) revealed two potential catalytic aspartate residues at positions 91 and 378 in the 601-amino acid-long CgYps1 enzyme (Fig. 2*A*). However, multiple-protein sequence alignment of CgYps7 with *S. cerevisiae* yapsins, including ScYps7, and *C. albicans* Saps, including Sap10, did not identify any conserved catalytic aspartate residues in the CgYps7 enzyme. Hence, we decided to mutate the predicted catalytic aspartate residues at positions 91 and 378 in CgYps1 only.

Through site-directed mutagenesis, two identified aspartate residues were individually converted to alanine, and the ability of mutant enzymes to complement the ion and low-pH sensitivity of the *Cgyps1–11*Δ mutant was tested. CgYps1 has previously been implicated in survival of acid stress and resistance to surplus metal ions under normal and pH 7.5 conditions (16, 18). As shown in Fig. 2*B*, growth defects of the *Cgyps1–11*Δ mutant in the pH 2.0 medium and the medium containing calcium chloride or zinc chloride could not be restored upon expression of the CgYps1 enzyme carrying D91A and D378A substitutions, indicating that both aspartic acid residues are required for cellular functions of CgYps1. As both Asp-91 and Asp-378 residues appear to be functionally important, CgYps1 carrying alanine in place of the aspartate at position 91 (CgYps1^D91A^) was used for further studies.

Next, we checked whether lower β-glucan and mannan levels, higher chitin content, and reduced biofilm formation in the *Cgyps1–11*Δ mutant could be restored by expression of the putative catalytically dead CgYps1, but we found no phenotypic complementation (Fig. 1, *C–F*). Together, these results suggest that the predicted catalytic aspartate residue at position 91 of the CgYps1 enzyme is required for maintenance of cell wall homeostasis, whereas aspartic acid residues, which contribute to the proteolytic activity of CgYps7, are yet to be identified.

### Response of human THP-1 macrophages to infection with C. glabrata WT and Cgyps1–11Δ mutant

**Figure 3.**
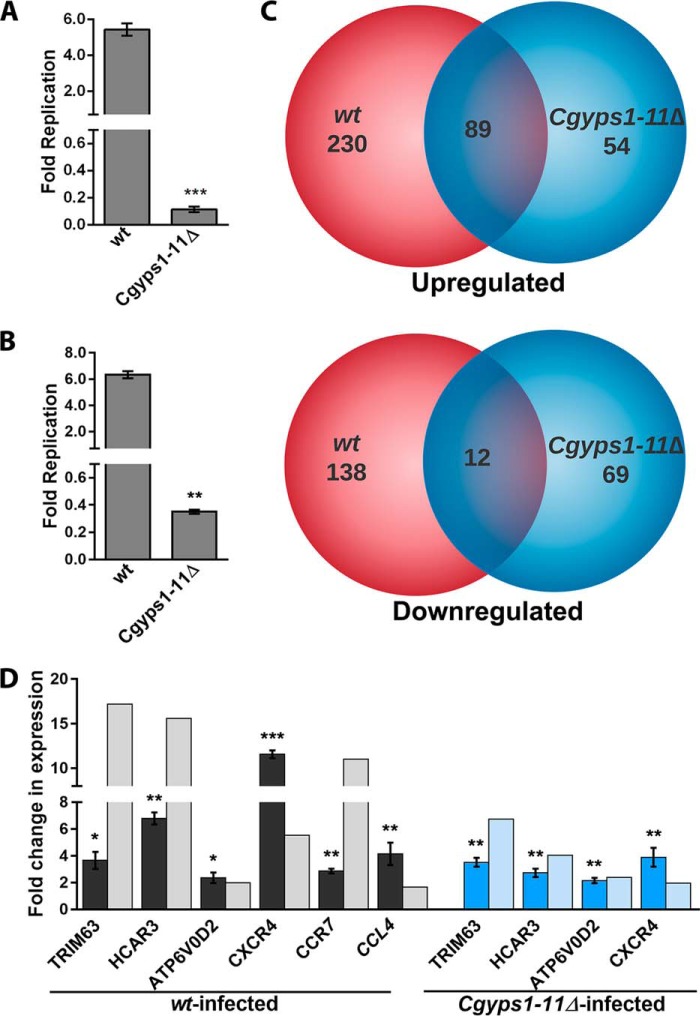
***C. glabrata* infection to THP-1 cells invokes a transcriptional response.** *A* and *B*, survival defect of the *Cgyps1–11*Δ mutant in macrophages. *C. glabrata* WT and *Cgyps1–11*Δ mutant strains were infected to PMA-differentiated THP-1 macrophages (*A*) and macrophages derived from human peripheral blood monocytes (*B*) at 10:1 MOI, and intracellular yeast cfu were measured by plating macrophage lysates 2 and 24 h postinfection. *Fold replication* indicates the ratio of the number of intracellular *C. glabrata* cells at 24 h to that at 2 h postinfection. Data represent mean ± S.E. (*error bars*) of 3–4 independent experiments. **, *p* < 0.01; ***, *p* < 0.001; unpaired two-tailed Student's *t* test. *C*, Venn diagram illustrating overlap in differentially expressed genes between WT– and *Cgyps1–11*Δ–infected THP-1 macrophages compared with uninfected cells. *D*, qPCR validation of microarray data. RNA was extracted using TRIzol from 6-h uninfected and WT- and *Cgyps1–11*Δ–infected THP-1 macrophages, and transcript levels of the indicated genes were measured by qPCR. Data (mean ± S.E., *n* = 3–4) were normalized against the *GAPDH* mRNA control and represent -fold change in expression in *C. glabrata*–infected macrophages compared with uninfected THP-1 macrophages. *, *p* < 0.05; **, *p* < 0.01; ***, *p* < 0.001; paired two-tailed Student's *t* test.

Cell wall polysaccharides have been implicated in activation of the host immune response both *in vitro* and *in vivo* (24–27). The above-described altered cell wall composition in the *Cgyps1–11*Δ mutant led us to examine the transcriptional response of macrophages, which represent the first line of the defense machinery of the host innate immune system (28, 29), toward infection with mutant cells. For infection studies, THP-1 macrophages were generated by phorbol ester treatment of the human monocytic cell line THP-1. The *Cgyps1–11*Δ mutant has previously been reported to be killed in murine and human macrophages (9, 12). Consistently, after 24 h co-culturing, only 15% of *Cgyps1-11*Δ cells were found to be viable in THP-1 macrophages, whereas WT cells displayed about 5-fold replication (Fig. 3*A*). Similarly, WT cells displayed 6-fold replication, whereas the *Cgyps1–11*Δ mutant exhibited only 35% survival in human blood monocyte-derived macrophages (Fig. 3*B*). These data indicate that CgYps1–11 are required for intracellular survival of *C. glabrata*.

Next, to investigate whether gene expression is different between WT- and *Cgyps1–11*Δ-infected macrophages, we determined the transcriptional response, through genome-wide microarray analysis, of THP-1 macrophages to infection with WT and mutant cells for 6 h. The 6-h time point was chosen, as the *Cgyps1–11*Δ mutant is known to retain viability in THP-1 macrophages for 8 h (12). Transcript profiles were compared between WT- or *Cgyps1–11*Δ-infected and uninfected THP-1 cells. A total of 469 and 224 genes were found to be differentially regulated in WT-infected and *Cgyps1–11*Δ-infected THP-1 cells, respectively (Fig. 3*C*). Genes were considered to be differentially regulated if -fold change in expression compared with uninfected phorbol 12-myristate 13-acetate (PMA)-treated THP-1 cells was >1.5-fold (*p* value <0.05). In WT-infected macrophages, 319 genes were up-regulated and 150 genes were down-regulated, whereas *Cgyps1–11*Δ-infected macrophages showed induction and repression of 143 and 81 genes, respectively (Fig. 3*C* and Tables S2 and S3). A set of 89 and 12 genes were common in up-regulated and down-regulated gene sets, respectively, between WT- and mutant-infected macrophages (Fig. 3*C*).

Transcriptional response of THP-1 cells to *C. glabrata* WT infection was largely represented by differential expression of genes involved in “chemotaxis,” “inflammatory response,” “regulation of GTPase activity,” “apoptosis,” and “signal transduction” (Table 1). An examination of the statistically significant enriched gene ontology terms for biological processes, using the DAVID functional annotation clustering tool, revealed many GO terms, including “negative regulation of cytokine secretion,” “negative regulation of inflammatory response,” “positive regulation of cytosolic calcium ion concentration,” and “ion transport” to be enriched in the up-regulated gene set. Further, GO process terms “tumor necrosis factor (TNF)-mediated signaling pathway,” “positive regulation of phosphatidylinositol 3-kinase (PI3K) signaling,” and “positive regulation of MAPK (mitogen-activated protein kinase) cascade” were found to be enriched in the down-regulated gene set of WT-infected macrophages (Table 1). These data suggest that THP-1 cells respond to *C. glabrata* infection by transcriptional induction of negative regulators of cytokine secretion and repression of MAPK, PI3K, and TNF signaling pathways.

**Table 1:** **GO-BP enrichment analysis of DEGs in *C. glabrata* WT– and *Cgyps1–11*Δ–infected THP-1 macrophages**

GO ID	GO term	*p*
**WT-infected THP-1 macrophages**		
Up-regulated DEGs		
GO:0006935	Chemotaxis	0.0001
GO:0007165	Signal transduction	0.0003
GO:0006954	Inflammatory response	0.0003
GO:0050710	Negative regulation of cytokine secretion	0.0008
GO:0070098	Chemokine-mediated signaling pathway	0.0009
GO:0050728	Negative regulation of inflammatory response	0.0015
GO:0051209	Release of sequestered calcium ion into cytosol	0.0038
GO:0001666	Response to hypoxia	0.0058
GO:0006955	Immune response	0.0067
GO:0007254	JNK cascade	0.0072
GO:0051491	Positive regulation of filopodium assembly	0.0076
GO:0043065	Positive regulation of apoptotic process	0.0079
GO:0043401	Steroid hormone mediated signaling pathway	0.0121
GO:0043547	Positive regulation of GTPase activity	0.0167
GO:0060644	Mammary gland epithelial cell differentiation	0.0170
GO:0060326	Cell chemotaxis	0.0189
GO:0007204	Positive regulation of cytosolic calcium ion concentration	0.0190
GO:0021794	Thalamus development	0.0196
GO:0005975	Carbohydrate metabolic process	0.0199
GO:0007049	Cell cycle	0.0213
GO:0050729	Positive regulation of inflammatory response	0.0276
GO:0006468	Protein phosphorylation	0.0280
GO:0010039	Response to iron ion	0.0317
GO:0001755	Neural crest cell migration	0.0333
GO:0045766	Positive regulation of angiogenesis	0.0347
GO:0007275	Multicellular organism development	0.0360
GO:0006629	Lipid metabolic process	0.0373
GO:0071345	Cellular response to cytokine stimulus	0.0460
GO:2000345	Regulation of hepatocyte proliferation	0.0463
GO:0006811	Ion transport	0.0496
Downregulated DEGs		
GO:0006954	Inflammatory response	0.0001
GO:0043410	Positive regulation of MAPK cascade	0.0003
GO:0060750	Epithelial cell proliferation involved in mammary gland duct elongation	0.0003
GO:0014068	Positive regulation of phosphatidylinositol 3-kinase signaling	0.0011
GO:0070374	Positive regulation of ERK1 and ERK2 cascade	0.0014
GO:0097190	Apoptotic signaling pathway	0.0015
GO:0010863	Positive regulation of phospholipase C activity	0.0017
GO:0045778	Positive regulation of ossification	0.0030
GO:0061036	Positive regulation of cartilage development	0.0054
GO:0048663	Neuron fate commitment	0.0061
GO:0060749	Mammary gland alveolus development	0.0061
GO:0048146	Positive regulation of fibroblast proliferation	0.0063
GO:0000187	Activation of MAPK activity	0.0067
GO:0071346	Cellular response to interferon-γ	0.0073
GO:1902895	Positive regulation of pri-miRNA transcription from RNA polymerase II promoter	0.0084
GO:0032092	Positive regulation of protein binding	0.0088
GO:0006898	Receptor-mediated endocytosis	0.0098
GO:0006935	Chemotaxis	0.0105
GO:0060326	Cell chemotaxis	0.0105
GO:0001938	Positive regulation of endothelial cell proliferation	0.0124
GO:0070098	Chemokine-mediated signaling pathway	0.0134
GO:0072104	Glomerular capillary formation	0.0139
GO:0055007	Cardiac muscle cell differentiation	0.0151
GO:0008284	Positive regulation of cell proliferation	0.0163
GO:0042981	Regulation of apoptotic process	0.0167
GO:0014066	Regulation of phosphatidylinositol 3-kinase signaling	0.0172
GO:0043065	Positive regulation of apoptotic process	0.0186
GO:0043552	Positive regulation of phosphatidylinositol 3-kinase activity	0.0196
GO:0048701	Embryonic cranial skeleton morphogenesis	0.0196
GO:0022414	Reproductive process	0.0208
GO:0072138	Mesenchymal cell proliferation involved in ureteric bud development	0.0208
GO:0003130	BMP signaling pathway involved in heart induction	0.0208
GO:0030501	Positive regulation of bone mineralization	0.0247
GO:0007188	Adenylate cyclase-modulating G-protein–coupled receptor signaling pathway	0.0274
GO:0060686	Negative regulation of prostatic bud formation	0.0276
GO:0045893	Positive regulation of transcription, DNA-templated	0.0276
GO:0008584	Male gonad development	0.0279
GO:0046854	Phosphatidylinositol phosphorylation	0.0279
GO:0002062	Chondrocyte differentiation	0.0302
GO:0007165	Signal transduction	0.0311
GO:0002138	Retinoic acid biosynthetic process	0.0344
GO:0061156	Pulmonary artery morphogenesis	0.0344
GO:0043949	Regulation of cAMP-mediated signaling	0.0344
GO:0060744	Mammary gland branching involved in thelarche	0.0344
GO:0002548	Monocyte chemotaxis	0.0346
GO:0001658	Branching involved in ureteric bud morphogenesis	0.0346
GO:0000165	MAPK cascade	0.0365
GO:0048015	Phosphatidylinositol-mediated signaling	0.0379
GO:0045944	Positive regulation of transcription from RNA polymerase II promoter	0.0406
GO:0045165	Cell fate commitment	0.0408
GO:0003151	Outflow tract morphogenesis	0.0408
GO:2001171	Positive regulation of ATP biosynthetic process	0.0411
GO:0003150	Muscular septum morphogenesis	0.0411
GO:0021978	Telencephalon regionalization	0.0411
GO:0002320	Lymphoid progenitor cell differentiation	0.0411
GO:0042487	Regulation of odontogenesis of dentin-containing tooth	0.0411
GO:0071356	Cellular response to tumor necrosis factor	0.0416
GO:0043547	Positive regulation of GTPase activity	0.0439
GO:0007219	Notch signaling pathway	0.0464
GO:0051782	Negative regulation of cell division	0.0478
GO:0060449	Bud elongation involved in lung branching	0.0478
GO:0045656	Negative regulation of monocyte differentiation	0.0478
GO:0060687	Regulation of branching involved in prostate gland morphogenesis	0.0478
GO:0033209	Tumor necrosis factor–mediated signaling pathway	0.0494

***Cgyps1–11*Δ–infected THP-1 macrophages**		
Up-regulated DEGs		
GO:0051607	Defense response to virus	0.0253
GO:0009615	Response to virus	0.0381
GO:0007420	Brain development	0.0395
GO:0006139	Nucleobase-containing compound metabolic process	0.0430
Down-regulated DEGs		
GO:0060412	Ventricular septum morphogenesis	0.0051
GO:0032729	Positive regulation of interferon-γ production	0.0125
GO:0001570	Vasculogenesis	0.0182
GO:0061156	Pulmonary artery morphogenesis	0.0183
GO:0007179	Transforming growth factor β receptor signaling pathway	0.0454
GO:0001916	Positive regulation of T cell–mediated cytotoxicity	0.0470
GO:0051145	Smooth muscle cell differentiation	0.0470
GO:0008584	Male gonad development	0.0472

In our microarray data sets, 5 and 10 genes belonging to the GO term “chemotaxis” were repressed and induced, respectively. This differentially regulated gene set was represented primarily by genes encoding CC and CXC chemokines and their receptors, which suggests that *C. glabrata* infection leads to a strong chemotactic cell migration response. Notably, *C. glabrata* cells have been postulated to induce increased migration of monocytes to the site of infection (30), and macrophages are considered as “Trojan horses” for *C. glabrata* (7, 30). Hence, it is possible that a strong chemotactic response generated by macrophages may dampen the recruitment of neutrophils, which possess the ability to kill *C. glabrata* cells (31).

A closer inspection of individual gene expressions revealed that genes implicated in the inflammatory response, *CXCR4*, *TLR7*, *CCRL2*, *CEBPB*, and *P2RX7*, were induced in THP-1 macrophages in response to infection with both WT and *Cgyps1–11*Δ cells, which is reflective of a common transcriptional response of THP-1 cells to *C. glabrata* infection. Of note, *C. albicans* infection is known to result in production of chemokines and their receptors in monocytes and THP-1 macrophages, including CCR7, CCL3 (MIP-1α), and CCL4 (MIP-1β) (32, 33), whereas the *CCL4* (MIP-1β) has previously been shown to be induced upon interaction of *C. glabrata* with polymorphonuclear cells (30). Moreover, up-regulation of the anti-apoptotic *BCL2* (B-cell lymphoma 2) gene in both WT*-* and mutant-infected macrophages is consistent with the earlier observation of lack of apoptosis in human monocyte-derived macrophages upon *C. glabrata* infection (10).

Next, we focused on differences in the gene expression pattern between WT– and *Cgyps1–11*Δ mutant–infected macrophages. Although they share a set of 89 common up-regulated genes, indicating that infection with both *C. glabrata* strains leads to a largely similar transcriptional induction response, the DAVID analysis for significant enriched GO terms suggested otherwise. Instead of GO terms “negative regulation of cytokine secretion” and “negative regulation of inflammatory response,” GO terms “defense response to virus,” “brain development,” and “nucleobase-containing compound metabolic process” were found to be enriched in *Cgyps1–11*Δ–infected macrophages (Table 1).

Further, a detailed analysis of differential gene expression pattern between WT– and *Cgyps1–11*Δ–infected macrophages revealed that of 15 “chemotaxis” genes (*PTAF4*, *PLAU*, *FPR1*, *CXCL1*, *CXCL16*, *CCRL2*, *CCR10*, *CCL1*, *CCL2*, *CCL20*, *CCL28*, *CCR7*, *CXCR3*, *CXCR4*, and *CXCR7*) differentially regulated in WT-infected macrophages, only two genes (*CXCR4* and *CCRL2*) were up-regulated in mutant-infected macrophages. Similarly, of seven up-regulated genes (*CXCR4*, *CCR7*, *GPER*, *ADM*, *SWAP70*, *CCR10*, and *CCL28*) that belong to the “positive regulation of cytosolic calcium ion concentration” GO term, only one gene (*CXCR4*) was up-regulated in mutant-infected macrophages. Furthermore, whereas genes involved in positive regulation of MAPK cascade, positive regulation of PI3K signaling, and TNF-mediated signaling pathways were down-regulated in WT-infected macrophages, genes implicated in ventricular septum morphogenesis, transforming growth factor β receptor signaling pathway, positive regulation of interferon-γ production, and vasculogenesis processes were down-regulated in *Cgyps1–11*Δ–infected macrophages (Table 1). Altogether, these data point toward a subdued and differential immune response invoked by the *Cgyps1–11*Δ mutant upon infection to THP-1 macrophages. It is also possible that the inability of the *Cgyps1–11*Δ mutant to repress the transcriptional machinery of key signaling cascades results in its death in THP-1 macrophages.

Last, to validate the microarray expression data, we performed qPCR analysis for highly induced *HCAR3*, *TRIM63*, *ATP6V0D2*, *CXCR4*, *CCR7*, and *CCL4* genes. *HCAR3*, *TRIM63*, *ATP6V0D2*, *CXCR4*, *CCR7*, and *CCL4* genes code for the hydroxycarboxylic acid receptor 3, E3 ubiquitin-protein ligase–containing tripartite motif, H^+^-transporting ATPase V0 subunit d2, chemokine (C*X*C motif) receptor 4, chemokine (CC motif) receptor 7, and chemokine (CC motif) ligand 4, respectively. The first four genes were up-regulated in WT– and *Cgyps1–11*Δ–infected macrophages, compared with uninfected macrophages, whereas the *CCR7* and *CCL4* genes were uniquely induced in WT-infected macrophages in the microarray experiment. In agreement, all six genes displayed the same expression pattern in the qPCR experiment (Fig. 3*D*).

Overall, our global gene expression analysis revealed that THP-1 cells respond to WT *C. glabrata* infection via differential expression of genes involved in inflammatory response, chemotaxis, regulation of GTPase activity, and chemokine-mediated signaling pathway (Table 1). On the contrary, mutant-infected macrophages primarily displayed up-regulation of viral response gene expression (Table 1). The observed differential gene expression between WT– and *Cgyps1–11*Δ–infected macrophages, however, was not simply due to lower internalization rates, as both strains were ingested at equal efficiency and displayed similar intracellular counts at 2 h postinfection. Thus, in all likelihood, differential gene expression patterns of WT– and *Cgyps1–11*Δ–infected THP-1 cells reflect the individual yeast strain's ability to elicit the macrophage immune response.

### THP-1 macrophages produce IL-1β in response to C. glabrata infection

Next, we examined whether differences in gene expression pattern between WT– and *Cgyps1–11*Δ–infected macrophages are mirrored in the release of pro- and anti-inflammatory cytokines. To test this, we measured the levels of IL-1α, IL-1β, IL-2, IL-4, IL-6, IL-8, IL-10, IL-12, IL-17A, IFN-γ, TNFα, and granulocyte-macrophage colony-stimulating factor (GM-CSF) in culture media of either uninfected THP-1 cells or THP-1 macrophages infected with WT and *Cgyps1–11*Δ cells for 24 h. Notably, infection of *C. glabrata*, unlike that of *S. cerevisiae* and *C. albicans*, is known to induce reduced production of IL-6, IL-8, TNFα, IL-1β, and IFN-γ in human monocyte-derived macrophages (10). However, GM-CSF production was robust upon *C. glabrata* infection (10).

**Figure 4.**
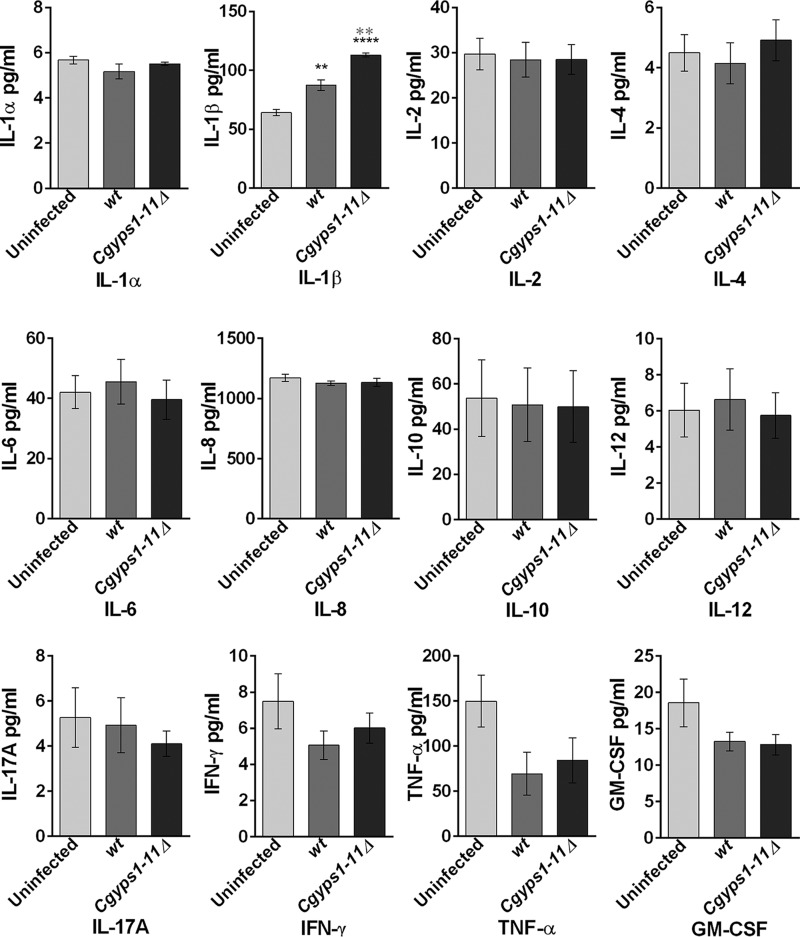
**CgYapsins are required to suppress the production of the pro-inflammatory cytokine IL-1β**. Shown is analysis of the indicated cytokines in uninfected and WT– and *Cgyps1–11*Δ–infected THP-1 macrophages. PMA-differentiated THP-1 cells were either left uninfected or infected with *C. glabrata* WT and *Cgyps1–11*Δ mutant for 24 h. Expression of cytokines was evaluated in 100 μl of culture medium using the Multi-Analyte ELISArray kit. Data represent mean ± S.E. (*error bars*) of 3–4 independent experiments. Statistically significant differences in IL-1β levels between uninfected and *C. glabrata*–infected and between WT– and *Cgyps1–11*Δ–infected macrophages are indicated by *black* and *gray asterisks*, respectively. **, *p* < 0.01, ****, *p* < 0.0001; unpaired two-tailed Student's *t* test.

As shown in Fig. 4, *C. glabrata* infection led to no significant production of any cytokine in THP-1 macrophages except for IL-1β. A 1.4-fold higher level of IL-1β was observed in WT-infected macrophages compared with uninfected macrophages (Fig. 4). Differential cytokine profiles of *C. glabrata*–infected THP-1 cells and monocyte-derived macrophages could be due to different type or nature (cultured *versus* primary) of the cells. *Cgyps1–11*Δ–infected macrophages released 2-fold higher amounts of IL-1β in the culture media compared with uninfected macrophages, and the difference in IL-1β secretion between WT– and *Cgyps1–11*Δ–infected macrophages was also statistically significant (Fig. 4). These results suggest that one function of CgYapsins is to keep IL-1β production and secretion in check in macrophages. As other proinflammatory cytokines, such as TNFα and IL-6, were not induced upon *C. glabrata* infection, we probed deeper to understand the basis for specific induction of IL-1β production.

IL-1β is a key mediator of the inflammatory response and is synthesized by activated macrophages as a pro-protein, which, upon proteolytic cleavage by the caspase 1 enzyme, is converted to its active form (34). *C. albicans* infection has previously been shown to result in pro-IL-1β production and activation of the NLRP3 inflammasome, which depends upon the spleen tyrosine kinase (Syk) (35). Syk also plays an important role in activated immunoglobulin Fc receptor signaling (36).

**Figure 5.**
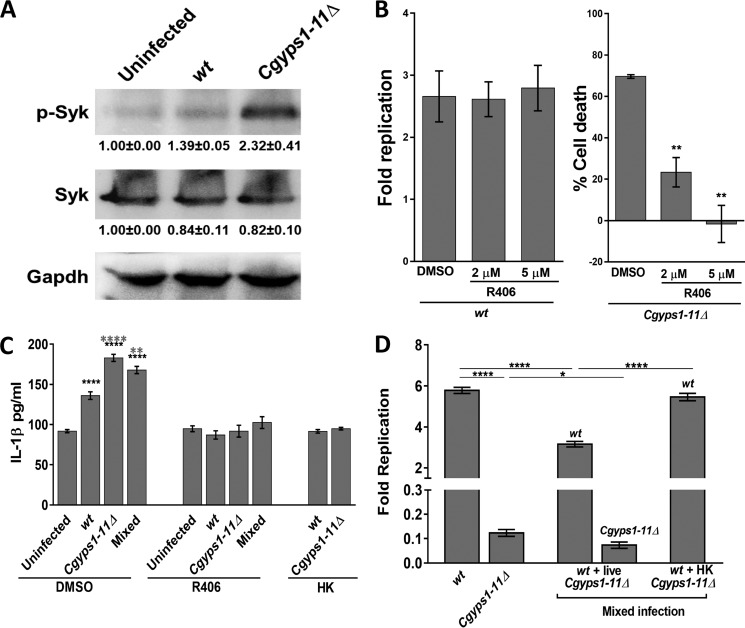
**Cgyps1–11Δ–induced IL-1**β **production in THP-1 macrophages depends on Syk.** *A*, representative immunoblot illustrating phosphorylated Syk in uninfected, and WT– and *Cgyps1–11*Δ–infected THP-1 macrophages. THP-1 cell extracts containing 120 μg of protein were resolved on 10% SDS-PAGE and probed with anti-phospho-Syk, anti-Syk, and anti-GAPDH antibodies. GAPDH was used as a loading control. For quantification, intensity of individual bands in three independent Western blots was measured using the ImageJ densitometry software. Total Syk and phosphorylated Syk signal in each lane was normalized to the corresponding GAPDH signal (considered as 1.0). Data (mean ± S.E. (*error bars*)) are presented as -fold change in signal intensity levels in infected samples compared with uninfected samples (taken as 1.0) *below* the blot. *B*, intracellular survival of WT and *Cgyps1–11*Δ mutant in R406-treated THP-1 macrophages. 2 and 5 μm R406 was added to THP-1 macrophages 2 h before *C. glabrata* infection, and infection was continued in the presence of R406. *Fold Replication* for WT cells indicates the ratio of the number of intracellular *C. glabrata* cells at 24 h to that at 2 h postinfection. Percentage cell death for the *Cgyps1–11*Δ mutant indicates viability loss of mutant cells in DMSO- and R406-treated THP-1 cells between 2 and 24 h of infection, as determined by measurement of intracellular cfu at these two time points. Data represent mean ± S.E. (*n* = 3). **, *p* < 0.01; unpaired two-tailed Student's *t* test. *C*, measurement of secreted IL-1β in DMSO- or R406-treated, *C. glabrata*–infected THP-1 cells. THP-1 macrophage infection was done as described in the legend to Fig. 5*B* with *C. glabrata* cells at an MOI of 1:1, and IL-1β levels in the culture supernatant were measured after 24 h using the human IL-1β ELISA Set II kit. *HK*, heat-killed dead *C. glabrata* cells that were obtained after incubation at 95 °C for 20 min. *Mixed infection*, co-infection of THP-1 cells with WT*-* and *Cgyps1–11*Δ cells. Notably, R406 treatment abolished *C. glabrata*–induced IL-1β production in THP-1 cells. Data (mean ± S.E.; *n* = 4–8) represent secreted IL-1β levels under the indicated conditions. Statistically significant differences in IL-1β levels between uninfected and *C. glabrata*–infected and between WT*-* and *Cgyps1–11*Δ–infected macrophages are indicated by *black* and *gray asterisks*, respectively. **, *p* < 0.01, ****, *p* < 0.0001; unpaired two-tailed Student's *t* test. *D*, assessment of intracellular proliferation of WT cells in THP-1 macrophages infected with live WT and live or dead *Cgyps1–11*Δ cells. The total number of *C. glabrata* cells infected to THP-1 macrophages was the same (1 × 10^5^) in both single and mixed infections. *Fold Replication* indicates the ratio of the number of intracellular *C. glabrata* cells at 24 h to that at 2 h postinfection. Data represent mean ± S.E. of 4–6 independent experiments. *, *p* < 0.05; ****, *p* < 0.0001; unpaired two-tailed Student's *t* test.

To determine the signaling pathway that contributes to elevated IL-1β production in *Cgyps1–11*Δ–infected THP-1 cells, we first checked whether Syk is activated in response to *C. glabrata* infection. We observed a 1.4- and 2.3-fold higher Syk phosphorylation in WT–infected and *Cgyps1–11*Δ–infected macrophages, respectively, compared with uninfected THP-1 cells (Fig. 5*A*), indicative of a hyperactivated Syk in *Cgyps1–11*Δ–infected macrophages. We next verified these results using the kinase inhibitor approach. R406, a specific, ATP-competitive inhibitor of Syk, is known to impede Syk-dependent Fc receptor–mediated activation of macrophages and *C. albicans*-mediated activation of the inflammasome (35, 36). We reasoned that if macrophage-mediated killing of *Cgyps1–11*Δ cells is due to increased IL-1β levels and hyperactive Syk, their inhibition should rescue mutant cell death in THP-1 cells. Hence, we treated WT– and *Cgyps1–11*Δ–infected THP-1 cells with R406 and determined intracellular growth profiles of yeast cells. We also checked whether R406 treatment had any effect on viability of THP-1 cells and found none. Whereas intracellular proliferation of *C. glabrata* WT cells remained unaffected by R406 treatment of THP-1 cells, viability loss of *Cgyps1–11*Δ cells was rescued in R406-treated macrophages in an R406 dose–dependent manner (Fig. 5*B*). Of note, *Cgyps1–11*Δ cells did not display any intracellular replication, as the number of intracellular mutant cfu was similar at 2 and 24 h after infection in R406 (5 μm)-treated cells. However, compared with 30% cells remaining viable in untreated THP-1 cells, 75–100% of *Cgyps1–11*Δ cells retained viability in R406-treated THP-1 macrophages after 24 h of co-incubation (Fig. 5*B*). These results suggest that Syk inhibition allows the *Cgyps1–11*Δ mutant to survive in THP-1 macrophages. These data also indicate that Syk signaling activation has no role in the control of intracellular proliferation *in vitro*, as *C. glabrata* WT cells exhibited similar increase in intracellular cfu in R406-treated and untreated THP-1 cells (Fig. 5*B*).

Next, to check whether R406 treatment dampens IL-1β production, we measured cytokine levels in R406-treated THP-1 macrophages. As observed earlier (Fig. 4), *C. glabrata* infection led to higher IL-1β production (Fig. 5*C*). However, we found no increase in secreted IL-1β in R406-treated, WT– and *Cgyps1–11*Δ–infected macrophages compared with R406-treated uninfected macrophages (Fig. 5*C*). Importantly, increase in IL-1β levels was also not observed when THP-1 macrophages were infected with heat-killed WT or *Cgyps1–11*Δ cells (Fig. 5*C*). Collectively, these results link rescue of the mutant viability loss with IL-1β levels in THP-1 cells and support the following four key conclusions. First, IL-1β production in THP-1 cells, upon *C. glabrata* infection, depends on Syk. Second, the Syk-dependent IL-1β production requires live *C. glabrata* cells. Third, CgYapsins are required to suppress this inflammatory response of THP-1 cells. Fourth, *Cgyps1–11*Δ cell death in THP-1 macrophages is, in part, due to activated Syk signaling and higher IL-1β production.

Further, we checked whether *Cgyps1–11*Δ-induced IL-1β production and macrophage activation had an effect on intracellular replication of WT *C. glabrata* cells. For this, we co-infected THP-1 cells with WT and *Cgyps1–11*Δ cells at the same time. In this mixed infection, WT and *Cgyps1–11*Δ cells were added in equal ratio (5 × 10^4^ cells/strain), and intracellular cfu of both strains were found to be similar after 2-h internalization by THP-1 macrophages. As shown in Fig. 5*D*, WT cells proliferated 5.6-fold when infected singly to THP-1 macrophages. In contrast, they displayed 40% less intracellular replication when co-infected with *Cgyps1–11*Δ mutant cells (Fig. 5*D*). This adverse effect on replication of WT cells depended upon the presence of live *Cgyps1–11*Δ cells, as WT cells proliferated 5.5-fold during the mixed infection of dead *Cgyps1–11*Δ and WT cells to THP-1 macrophages (Fig. 5*D*). Of note, THP-1 cells co-infected with live WT and *Cgyps1–11*Δ cells secreted 23% more IL-1β compared with WT strain–infected macrophages (Fig. 5*C*). This increase depended on Syk activation, as R406-treated macrophages showed no increase in IL-1β levels upon co-infection with WT and *Cgyps1–11*Δ cells (Fig. 5*C*). Of note, the decreased intracellular replication of WT cells in mixed live strain macrophage infection is unlikely to be solely due to increased IL-1β production and secretion, as R406-dependent reduction in IL-1β levels earlier had no effect on WT proliferation (Fig. 5*B*). Importantly, the *Cgyps1–11*Δ mutant could not survive in THP-1 cells, regardless of whether the infection was mixed or single (Fig. 5*D*). However, the mutant did show 50% lower survival in mixed infections (Fig. 5*D*), whose molecular basis is yet to be determined.

**Figure 6.**
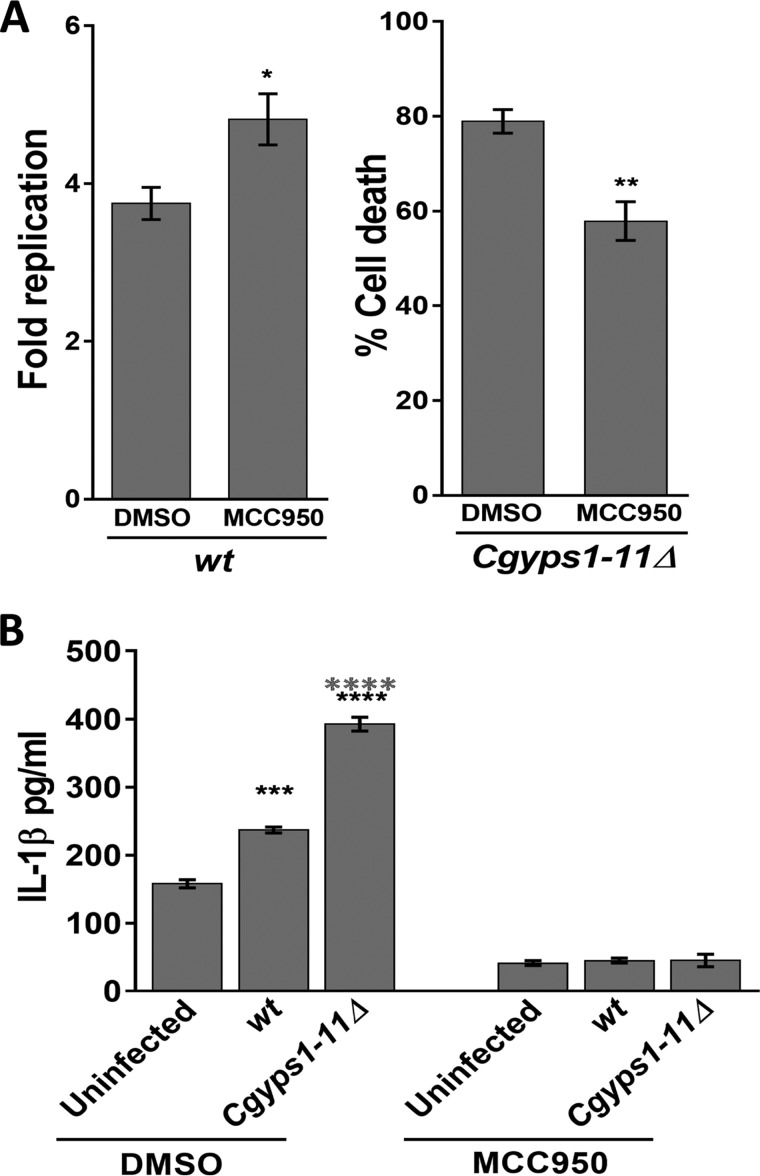
**NLRP3 inflammasome activation is required for *C. glabrata*-evoked IL-1β production in THP-1 macrophages.** *A*, intracellular survival of WT and *Cgyps1–11*Δ mutant in MCC950-treated THP-1 macrophages. 15 μm MCC950 was added to THP-1 macrophages 2 h before *C. glabrata* infection, and infection was continued in the presence of MCC950. *Fold Replication* for WT cells indicates the ratio of the number of intracellular *C. glabrata* cells at 24 h to that at 2 h postinfection. Percentage cell death for the *Cgyps1–11*Δ mutant indicates viability loss of mutant cells in DMSO- and MCC950-treated THP-1 cells between 2 and 24 h of infection, as determined by measurement of intracellular cfu at these two time points. Data represent mean ± S.E. (*error bars*) (*n* = 3). *, *p* < 0.05; **, *p* < 0.01; unpaired two-tailed Student's *t* test. *B*, measurement of secreted IL-1β in DMSO- or MCC950 (15 μm)-treated, *C. glabrata*–infected THP-1 cells. THP-1 macrophage infection was done as described in the legend to Fig. 5*B* with *C. glabrata* cells at an MOI of 1:1, and IL-1β levels in the culture supernatant were measured after 24 h. Data (mean ± S.E.; *n* = 3–4) represent secreted IL-1β levels under the indicated conditions. Statistically significant differences in IL-1β levels between uninfected and *C. glabrata*–infected and WT– and *Cgyps1–11*Δ–infected macrophages are indicated by *black* and *gray asterisks*, respectively. ***, *p* < 0.001; ****, *p* < 0.0001; unpaired two-tailed Student's *t* test.

Last, we checked whether IL-1β production depends upon activation of the NLRP3 inflammasome. For this, we used the inhibitor MCC950, which specifically blocks NLRP3 activation (37), and first monitored intracellular growth of *C. glabrata* in THP-1 macrophages treated with MCC950. Compared with DMSO-treated THP-1 cells, WT and *Cgyps1–11*Δ cells displayed 1.3-fold higher replication and 2.0-fold better survival, respectively, in MCC950-treated THP-1 macrophages (Fig. 6*A*). Next, we measured *C. glabrata*–invoked IL-1β production, and found that the MCC950 treatment completely abolished IL-1β production in THP-1 cells (Fig. 6*B*). These data implicate the NLRP3 inflammasome in IL-1β production and control of *C. glabrata* infection.

Altogether, our results suggest that enhanced production and secretion of IL-1β is likely to be deleterious to the survival of *C. glabrata* in macrophages, and fungal cell surface–associated aspartyl proteases are pivotal to the regulation of synthesis and/or secretion of this pro-inflammatory cytokine.

### C. glabrata infection invokes IL-1β production in the mouse model of systemic candidiasis

**Figure 7.**
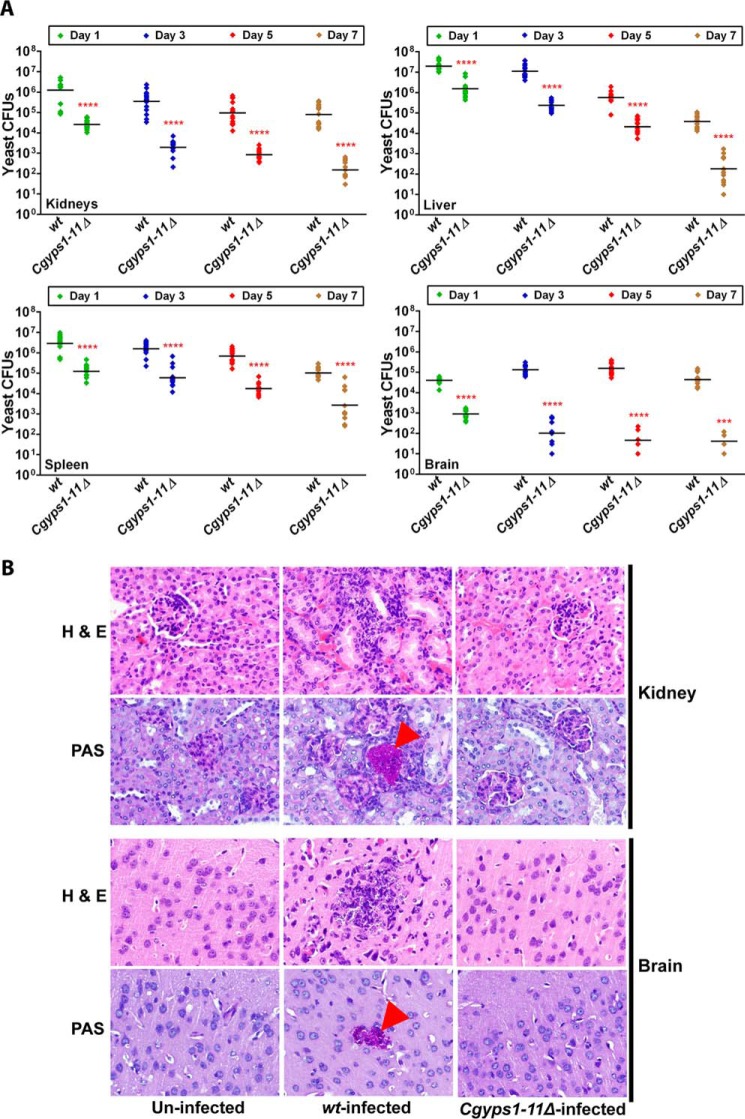
**CgYapsins are required for colonization and dissemination to the brain in the mouse model of systemic candidiasis.** *A*, kinetics of infection of *C. glabrata* WT and *Cgyps1–11*Δ cells in BALB/c mice. Mice were infected intravenously and sacrificed at the indicated days, and fungal burden in kidneys, liver, spleen, and brain was determined using a cfu-based assay. *Diamonds*, yeast cfu recovered from organs of the individual mouse; *horizontal line*, cfu geometric mean (*n* = 12–16) for each strain. Statistically significant differences in cfu between WT*-* and *Cgyps1–11*Δ-infected mice are *marked* (***, *p* < 0.001; ****, *p* < 0.0001; Mann–Whitney test). Of note, we could retrieve *Cgyps1–11*Δ cfu from the brain of only four mice of 14 infected animals 7 dpi. *B*, representative photomicrographs of hematoxylin and eosin– and PAS–stained kidney and brain sections from uninfected, WT-infected, and *Cgyps1–11*Δ–infected mice on day 3 after infection. Magnification was ×40. The *arrowheads* in tissue sections indicate PAS-positive yeast cells.

The *Cgyps1–11*Δ mutant–induced enhanced production of IL-1β in THP-1 macrophages prompted us to examine its effects *in vivo*. The *Cgyps1–11*Δ mutant is reported to be highly attenuated for virulence, after 7 days of infection, in the murine model of disseminated candidiasis, wherein organ fungal burden was taken as the end point (9). As the role of CgYapsins in colonization and dissemination of *C. glabrata* cells is yet to be understood, we decided to study the kinetics of *C. glabrata* infection in the mouse model of systemic candidiasis before any cytokine detection analysis. To achieve our goal, we injected female BALB/c mice with 4 × 10^7^ cells of either WT or the *Cgyps1–11*Δ mutant strain through the tail vein and assessed fungal burden in four organs, kidneys, liver, spleen, and brain, at 1, 3, 5, and 7 days postinfection (dpi). As shown in Fig. 7*A*, at day 1, mouse kidneys were colonized with 1.2 × 10^6^ WT cells, whereas only 2.6 × 10^4^ *Cgyps1–11*Δ cells represented renal fungal burden in the *Cgyps1–11*Δ–infected mice. Similarly, 13–45-fold lower cfu were recovered from other organs of the *Cgyps1–11*Δ–infected mice compared with the WT-infected mice 1 dpi (Fig. 7*A*), indicating that CgYapsins are required for the initial colonization and dissemination of *C. glabrata* cells. As the time course progressed, there was a constant decrease in the number of yeast cells that were harvested from kidneys, liver, and spleen of the WT-infected mice (Fig. 7*A*). These results preclude any significant multiplication of *C. glabrata* cells in these mouse organs.

Strikingly, the fungal burden in brain at 3 dpi was 3-fold higher than that observed at 1 dpi (Fig. 7*A*), suggesting that *C. glabrata* WT cells either take longer to reach the brain or undergo multiplication in the brain. Brain cfu for the WT strain remained similar between 3 and 5 dpi, whereas a 2-fold decrease was observed 7 dpi (Fig. 7*A*). Histological analysis of periodic acid–Schiff (PAS)-stained kidney and brain sections of WT-infected mice 3 dpi revealed the presence of *C. glabrata* cells in the form of a microcolony (Fig. 7*B*). However, no appreciable histological changes were observed in hematoxylin and eosin–stained tissue sections from WT– and *Cgyps1–11*Δ–infected mice compared with uninfected control mice (Fig. 7*B*). Consistent with an earlier report (11), these data suggest that *C. glabrata* infection does not lead to any gross abnormality in the tissue architecture.

Notably, *Cgyps1–11*Δ cells failed to colonize and/or migrate to the brain in substantial numbers, with a drastic decline in the mutant number at later time points (Fig. 7*A*). In fact, of 14 *Cgyps1–11*Δ–infected mice, mutant cells were recovered from the brain of only four mice 7 dpi (Fig. 7*A*). The *Cgyps1–11*Δ mutant did not fare well in other organs either, with mouse organ fungal burden decreasing sharply (Fig. 7*A*). These findings were corroborated with histological analysis, wherein no yeast colonies were observed in kidney and brain sections of the *Cgyps1–11*Δ–infected mice 3 dpi (Fig. 7*B*). Notably, PAS-stained kidney and brain sections of WT-infected mice did reveal the presence of yeast colonies (Fig. 7*B*). Together, these data suggest that CgYapsins are required for colonization, dissemination, and persistence on prolonged infection of *C. glabrata* in the brain, kidneys, liver, and spleen as well as for plausible replication in the brain during early stages of infection. These results are consistent with an earlier study reporting the CgYapsin requirement for survival in kidneys, liver, and spleen 7 dpi during systemic infections of mice (9).

**Figure 8.**
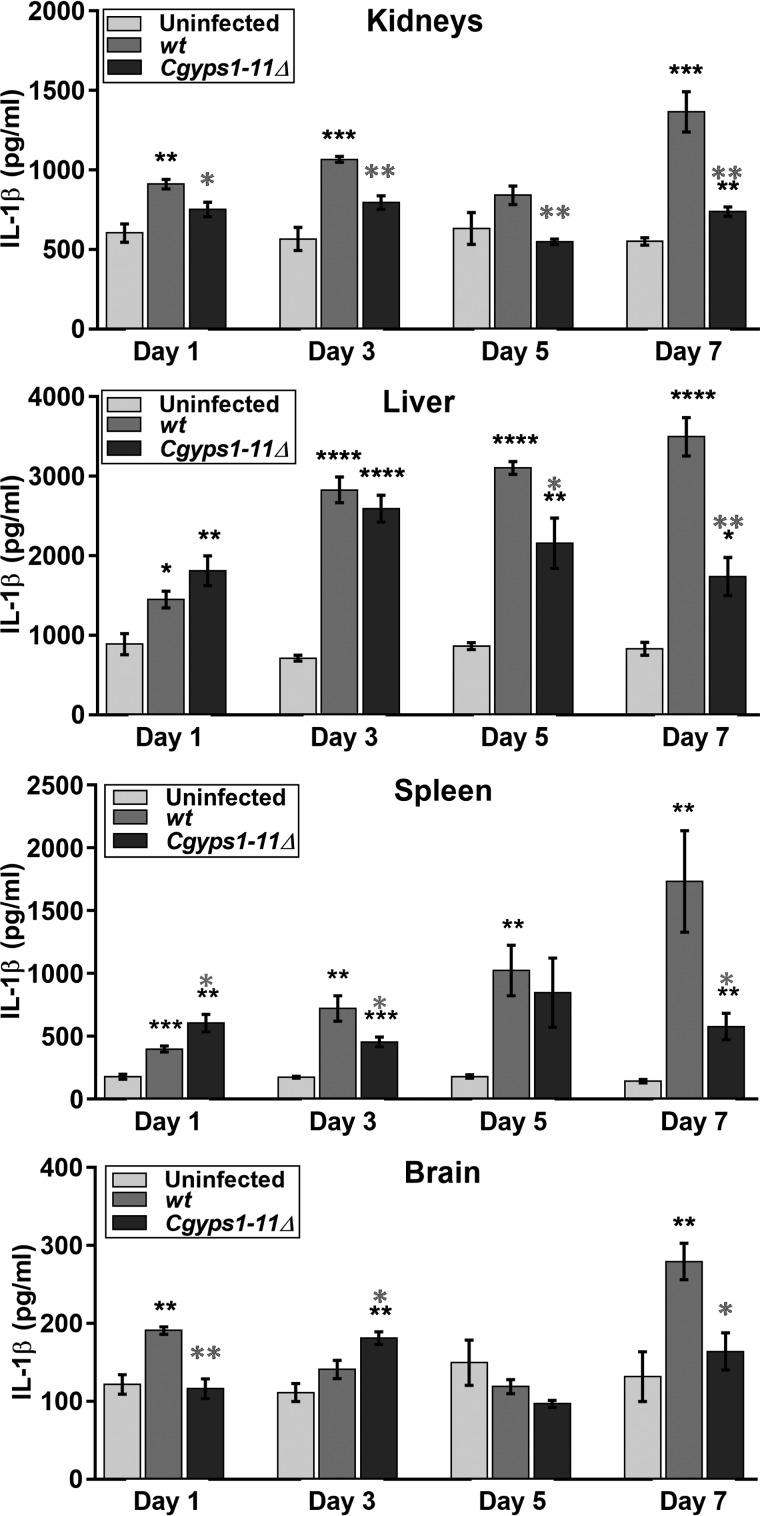
***C. glabrata* infection invokes IL-1 β production in the mouse model of systemic candidiasis.** Measurement of secreted IL-1β in tissue homogenates of kidneys, liver, spleen, and brain of mice infected with WT or *Cgyps1–11*Δ cells at the indicated days using the mouse IL-1β/IL-1F2 DuoSet ELISA kit. Data (mean ± S.E. (*error bars*), *n* = 4) represent IL-1 β levels under the indicated conditions. Statistically significant differences in IL-1β levels between uninfected and *C. glabrata*–infected, and WT– and *Cgyps1–11*Δ–infected mice are indicated by *black* and *gray asterisks*, respectively. *, *p* < 0.05; **, *p* < 0.01; ***, *p* < 0.001; ****, *p* < 0.0001; unpaired two-tailed Student's *t* test.

Next, to examine whether rapid clearance of the *Cgyps1–11*Δ mutant from mouse organs is due to induction of the inflammatory response, we measured levels of the cytokine IL-1β in tissue homogenates at 1, 3, 5, and 7 dpi. Enhanced IL-1β production was observed in all organs in WT-infected mice, whereas IL-1β levels were higher only in liver and spleen of the *Cgyps1–11*Δ–infected mice at 1 dpi (Fig. 8). Further, although IL-1β levels varied at later time points, significantly higher (2–12-fold) IL-1β amounts were still observed 7 dpi in kidneys, liver, spleen, and brain of WT–infected mice compared with uninfected mice (Fig. 8). Notably, IL-1β levels were lower overall in all organs of the *Cgyps1–11*Δ–infected mice compared with WT-infected mice at 7 dpi (Fig. 8), which appears to correlate partially with the mutant cfu burden in organs. These findings suggest that regulated production and release of IL-1β occurs probably to control *C. glabrata* infection *in vivo*; however, inability of the *Cgyps1–11*Δ mutant to survive in mice is not due to increased production of IL-1β in tissues.

### Overexpression of CgYps4, -5, -8, and -10 partially rescues intracellular survival defect of the Cgyps1–11Δ mutant

**Figure 9.**
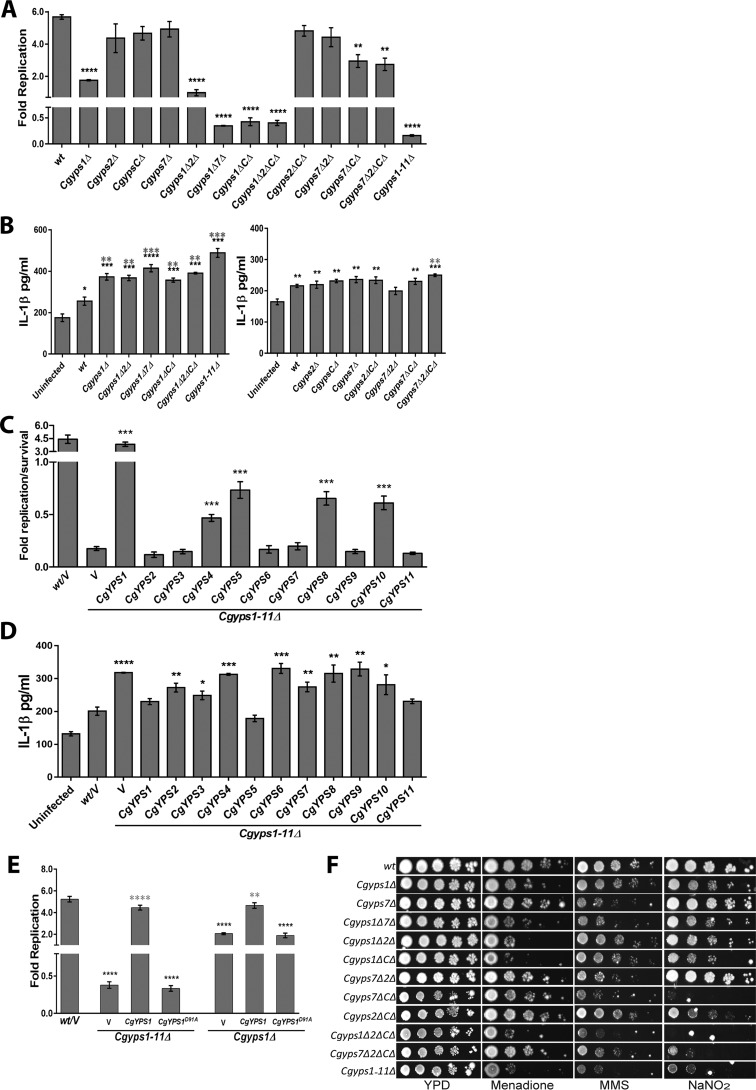
**CgYPS-C genes are required for survival of the macrophage internal milieu and nitrosative stress.** *A*, intracellular growth profiles of the indicated *Cgyps*Δ mutants in THP-1 macrophages. THP-1 macrophage infection was done as described in the legend to Fig. 3*A* with *C. glabrata* cells at an MOI of 10:1. *Fold Replication* indicates the ratio of the number of intracellular *C. glabrata* cells at 24 h to that at 2 h postinfection. Data represent mean ± S.E. (*error bars*) of 3–5 independent experiments. **, *p* < 0.01; ****, *p* < 0.0001; unpaired two-tailed Student's *t* test. *B*, IL-1β was measured in the culture supernatant of uninfected THP-1 and THP-1 cells infected with the indicated *C. glabrata* strains as described in the legend to Fig. 6*B*. Data (mean ± S.E.; *n* = 3–5) represent secreted IL-1β levels under the indicated conditions. Statistically significant differences in IL-1β levels between uninfected and *C. glabrata*–infected and between WT– and *Cgyps1–11*Δ–infected macrophages are indicated by *black* and *gray asterisks*, respectively. *, *p* < 0.05; **, *p* < 0.01; ***, *p* < 0.001; ****, *p* < 0.0001; unpaired two-tailed Student's *t* test. *C*, intracellular growth profiles of the *Cgyps1–11*Δ mutant overexpressing individual *CgYPS1–11* genes in THP-1 macrophages. *Fold Replication* indicates the ratio of the number of intracellular *C. glabrata* cells at 24 h to that at 2 h postinfection. Data represent mean ± S.E. of 3–5 independent experiments. *V*, *C. glabrata* strains carrying empty vector. ***, *p* < 0.001; unpaired two-tailed Student's *t* test. *D*, IL-1β was measured in the culture supernatant of uninfected THP-1 and THP-1 cells infected with the indicated *C. glabrata* strains as described in the legend to Fig. 6*B*. Data (mean ± S.E.; *n* = 4) represent secreted IL-1β levels under the indicated conditions. *V*, *C. glabrata* strains carrying empty vector. Statistically significant differences in IL-1β levels between WT– and *Cgyps1–11*Δ strain–infected macrophages are marked. *, *p* < 0.05; **, *p* < 0.01; ***, *p* < 0.001; ****, *p* < 0.0001; unpaired two-tailed Student's *t* test. *E*, intracellular survival of *Cgyps1*Δ and *Cgyps1–11*Δ mutant expressing either CgYps1 or putative catalytically dead CgYps1^D91A^ in THP-1 macrophages. *Fold Replication* indicates the ratio of the number of intracellular *C. glabrata* cells at 24 h to that at 2 h postinfection. Data represent mean ± S.E. of 3–7 independent experiments. *Black asterisks*, statistically significant differences between WT and mutants carrying either vector or CgYps1^D91A^ protein. *Gray asterisks*, statistically significant differences between mutants carrying vector and CgYps1 protein. **, *p* < 0.01; ****, *p* < 0.0001; unpaired two-tailed Student's *t* test. *F*, serial dilution spotting analysis of *Cgyps*Δ mutants under the indicated conditions. Menadione, MMS, and sodium nitrite (NaNO_2_) were used at a final concentration of 50 mm, 0.04%, and 60 mm, respectively. Growth was recorded after 2 days of incubation at 30 °C.

All intracellular survival and replication studies, so far, were carried out with the *Cgyps1–11*Δ mutant, which lacks all 11 CgYapsins. Therefore, next, to investigate which of these yapsin proteins is crucial to survival of *C. glabrata* cells in the host, we conducted macrophage and BALB/c mouse infection studies with mutants deleted for single, double, and multiple CgYps-encoding genes. Analysis of intracellular replication of different strains revealed that in addition to the 6.2-fold decline in viability of the *Cgyps1–11*Δ mutant, *Cgyps1*Δ*7*Δ, *Cgyps1*Δ*C*Δ, and *Cgyps1*Δ*2*Δ*C*Δ mutants exhibited 2.5–3.0-fold lower survival in THP-1 macrophages compared with 100% viability of the respective mutant strain at 2 h (Fig. 9*A*). These data indicate that lack of CgYps1 in conjunction with CgYps7, CgYpsC, and CgYps2&C affected intracellular survival of *C. glabrata*. In contrast, deletion of *CgYPS7* either alone or in conjunction with *CgYPS2* had no effect on intracellular proliferation (Fig. 9*A*). However, *Cgyps7*Δ*C*Δ and *Cgyps7*Δ*2*Δ*C*Δ mutants replicated 2-fold slower in THP-1 macrophages, compared with WT cells (Fig. 9*A*). Similarly, disruption of *CgYPS2* in the *Cgyps1*Δ background led to 1.8-fold less replication compared with *Cgyps1*Δ cells (Fig. 9*A*). Interestingly, *Cgyps2*Δ, *CgypsC*Δ, and *Cgyps2*Δ*C*Δ mutants displayed WT-like intracellular proliferation (Fig. 9*A*). Further, compared with WT-infected macrophages, enhanced IL-1β production was seen in THP-1 macrophages infected with *Cgyps1*Δ, *Cgyps1*Δ*2*Δ, *Cgyps1*Δ*7*Δ, *Cgyps1*Δ*C*Δ, *Cgyps1*Δ*2*Δ*C*Δ, *Cgyps7*Δ*2*Δ*C*Δ, and *Cgyps1–11*Δ mutants (Fig. 9*B*), which is in partial correlation with the intracellular survival pattern of *Cgyps*Δ mutants. Overall, these data indicate a role for *CgYPS-C* (*CgYPS3–6* and *CgYPS8–11*) genes in intracellular survival and/or replication of *C. glabrata*, as disruption of *CgYPC-C* genes adversely affected the survival and replication of *Cgyps1*Δ and *Cgyps7*Δ mutant, respectively. These results, consistent with findings of our earlier study in J774A.1 murine macrophages (9), suggest that *C. glabrata* cells do not replicate efficiently in the absence of CgYps1.

To further corroborate our results, we cloned and ectopically expressed all 11 *CgYPS* genes from an intermediate-strength promoter, *CgHHT2*, in the *Cgyps1–11*Δ mutant and examined intracellular survival. We found that ectopic expression of *CgYPS1* led to 4.5-fold replication of the *Cgyps1–11*Δ mutant, whereas ectopic expression of *CgYPS4*, -*5*, -*8*, and -*10* genes could partially rescue the viability loss of the *Cgyps1–11*Δ mutant in THP-1 macrophages (Fig. 9*C*). Importantly, *CgYPS7* overexpression had no positive effect on survival of the *Cgyps1–11*Δ mutant (Fig. 9*C*), thereby precluding any role for CgYps7 in intracellular survival of *C. glabrata in vitro*. Interestingly, overexpression of *CgYPS1*, *CgYPS5*, and *CgYPS11* in *Cgyps1–11*Δ cells evoked WT*-*like IL-1β production in THP-1 macrophages upon infection (Fig. 9*D*). As the *CgYPS11-*overexpressing *Cgyps1–11*Δ strain could not survive in THP-1 cells (Fig. 9*C*), the effect of *CgYPS11* on IL-1β expression in THP-1 cells needs to be further examined.

Overall, gene overexpression results correlate well with mutant infection studies, wherein disruption of eight *YPS-C* (*CgYPS3–6* and *CgYPS8–11*) genes adversely affected intracellular proliferation and survival of *Cgyps7*Δ and *Cgyps1*Δ mutant, respectively (Fig. 9*A*). These results also assign a function to *CgYPS4*, -*5*, -*8*, and -*10* genes individually, for the first time, in the pathobiology of *C. glabrata*.

As described above, of 11 CgYapsins, CgYps1 appears to be the key modulator of intracellular replication of *C. glabrata* cells (Fig. 9*A*). Hence, we next checked whether the predicted catalytic aspartate residue at position 91 of CgYps1 is required for survival in macrophages. For this, we measured intracellular survival and replication of *Cgyps1*Δ and *Cgyps1–11*Δ mutants expressing either *CgYPS1* or *CgYPS1^D91A^* allele. Expectedly, we found good complementation of mutant defects with CgYps1 expression (Fig. 9*E*). However, the putative catalytically dead CgYps1 could neither rescue the survival nor the proliferation defect of *Cgyps1–11*Δ and *Cgyps1*Δ mutant, respectively (Fig. 9*E*), suggesting that the predicted catalytic aspartate residue at position 91 of CgYps1 is necessary for intracellular survival and replication of *C. glabrata* in THP-1 macrophages.

Last, we examined whether intracellular behavior of *Cgyps*Δ mutants could be mimicked *in vitro* in the presence of a stressor likely to be encountered in the macrophage internal milieu. Macrophages are known to generate reactive oxygen and nitrogen species in response to microbial pathogens that may cause DNA damage (38). Hence, we checked growth of different *Cgyps*Δ mutants in the presence of the DNA-damaging agent methyl methane sulfonate (MMS) and nitrosative and oxidative stress–causing agents, sodium nitrite and menadione, respectively. We found mutants lacking *CgYPS1* and *CgYPS7* to be sensitive to menadione and MMS, respectively (Fig. 9*F*). Intriguingly, disruption of *CgYPS-C* genes in *Cgyps1*Δ, *Cgyps7*Δ, *Cgyps1*Δ*2*Δ, and *Cgyps7*Δ*2*Δ mutants led to significantly attenuated growth in the medium-containing sodium nitrite (Fig. 9*F*), suggesting that *CgYPS-C* genes contribute to survival under nitrosative stress conditions. In this context, it is noteworthy that six of eight *CgYPS-C* genes (*CgYPS4–5* and *CgYPS8–11*) were up-regulated upon internalization by murine J774A.1 macrophages, and disruption of *CgYPS-C* and *CgYPS2* genes in the *Cgyps1*Δ*7*Δ mutant significantly increased the nitric oxide production in J774A.1 cells compared with the *Cgyps1*Δ*7*Δ double mutant (9).

### CgYPS2 and CgYPS-C genes are required for survival in the murine model of systemic candidiasis

**Figure 10.**
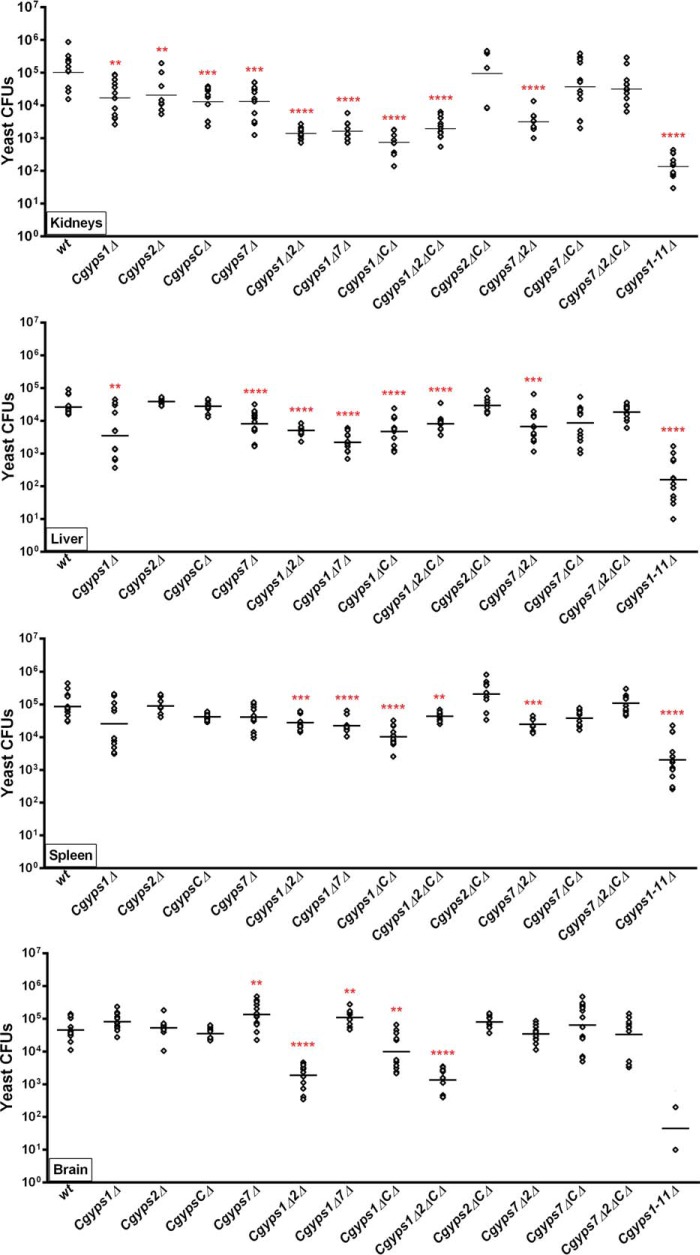
**CgYps2 and CgYps-C proteins are required for survival in the murine systemic candidiasis model.** BALB/c mice were infected intravenously with WT and the indicated *Cgyps*Δ mutants. At 7 dpi, mice were sacrificed, and organ fungal burden was calculated via a cfu assay. *Diamonds*, yeast cfu recovered from organs of the individual mouse; *horizontal line*, cfu geometric mean (*n* = 8–14) for each strain. Statistically significant differences between cfu recovered from WT– and *Cgyps*Δ–infected mice are marked (**, *p* < 0.01; ***, *p* < 0.001; ****, *p* < 0.0001; Mann–Whitney test).

Last, to delineate the role of *CgYPS-C* genes more precisely in virulence, we performed systemic infection studies in BALB/c mice with a panel of single, double, and multiple *Cgyps*Δ mutants (*Cgyps1*Δ, *Cgyps2*Δ, *CgypsC*Δ, *Cgyps7*Δ, *Cgyps1*Δ*2*Δ, *Cgyps1*Δ*7*Δ, *Cgyps1*Δ*C*Δ, *Cgyps1*Δ*2*Δ*C*Δ, *Cgyps2*Δ*C*Δ, *Cgyps7*Δ*2*Δ, *Cgyps7*Δ*C*Δ, *Cgyps7*Δ*2*Δ*C*Δ, and *Cgyps1–11*Δ). We found mice infected with all mutant strains, but for mice infected with *Cgyps2*Δ*C*Δ, *Cgyps7*Δ*C*Δ, *Cgyps7*Δ*2*Δ*C*Δ mutants exhibited less renal fungal burden compared with the WT-infected mice at 7 dpi (Fig. 10). In contrast, fewer cfu were retrieved from spleen of the mice infected with *Cgyps1*Δ*2*Δ, *Cgyps1*Δ*7*Δ, *Cgyps1*Δ*C*Δ, *Cgyps1*Δ*2*Δ*C*Δ, *Cgyps7*Δ*2*Δ, and *Cgyps1–11*Δ mutants only (Fig. 10). Mice infected with all other mutants, including single *Cgyps1*Δ, *Cgyps2*Δ, and *Cgyps7*Δ mutants, displayed WT-like cfu in spleen (Fig. 10). These data indicate an organ-specific role of CgYapsins in survival in mice and were further strengthened by the fungal burden data of liver and brain. Whereas mice infected with all *Cgyps*Δ mutants, except for *Cgyps2*Δ, *CgypsC*Δ, *Cgyps2*Δ*C*Δ, *Cgyps7*Δ*2*Δ, and *Cgyps7*Δ*2*Δ*C*Δ mutants, had less fungal burden in the liver (Fig. 10), only mice infected with *Cgyps1*Δ*2*Δ, *Cgyps1*Δ*C*Δ, *Cgyps1*Δ*2*Δ*C*Δ, and *Cgyps1–11*Δ strains showed less yeast cfu in the brain. Intriguingly, mice infected with mutants lacking *CgYPS7* either singly or in combination with *CgYPS1* showed higher fungal burden in the brain (Fig. 10). This was an unexpected result and could either imply a direct negative effect of CgYps7 on brain colonization and/or persistence or overexpression of factors in the *Cgyps7*-null mutant background that promote survival in the brain. Further investigations are currently under way to examine these two hypotheses.

Overall, the organ fungal burden data are consistent with the previous report indicating a major role for CgYps1 and CgYps7 in survival in mice and demonstrating that the *Cgyps2*Δ*C*Δ mutant is not attenuated for virulence (9). However, our data also unravel four new findings. First, lack of CgYps7 facilitates survival in brain. Second, CgYps2 and CgYps-C are required for survival in kidney. Third, absence of either *CgYPS2* or *CgYPS-C* genes has a strong negative effect on survival of the *Cgyps1*Δ mutant in kidney, liver, and spleen, which is similar in magnitude to that of the *CgYPS7* deletion. Last, loss of CgYps-C reverses the survival defects of *Cgyps7*Δ and *Cgyps7*Δ*2*Δ mutants in kidney and liver, and kidney, liver, and spleen, respectively. Altogether, our data yield new insights into the role of CgYps2, CgYps7, and CgYps-C in organ-specific survival of *C. glabrata* during systemic infections.

## Discussion

**Figure 11.**
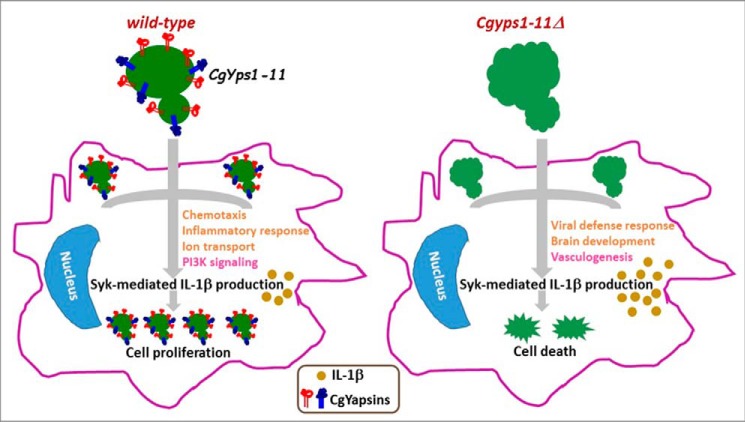
**Role of CgYapsins in interaction with macrophages.** Shown is a schematic model illustrating the role of CgYapsins in regulating the interaction of *C. glabrata* with THP-1 macrophages. CgYapsins modulate the macrophage transcriptional response probably to facilitate intracellular survival of *C. glabrata* through regulated production of Syk-dependent IL-1β. Lack of CgYapsins leads to increased IL-1β levels, which results in cell death. Altered cell wall attributes of the *Cgyps1–11*Δ mutant are depicted through *surface ridges*. Transcriptionally up-regulated and down-regulated processes in infected THP-1 cells are indicated in *light brown* and *pink*, respectively.

CgYapsins are pivotal to pathogenesis of *C. glabrata* (9). The *Cgyps1–11*Δ mutant, which lacks all 11 GPI-linked aspartyl proteases, is killed in macrophages and severely attenuated for virulence in the mouse systemic candidiasis model (9). Here, we examined how CgYapsins contribute to virulence and identify one mechanism by which they modulate the macrophage antifungal response. We demonstrated the *Cgyps1–11*Δ mutant to have altered cell wall polysaccharide attributes, which probably led, in part, to elicitation of an altered transcriptional response from THP-1 macrophages. Furthermore, *Cgyps1–11*Δ–infected macrophages produced relatively high levels of IL-1β in a Syk-signaling dependent manner, which contributed to intracellular killing of the mutant. These data reveal two key findings. First, macrophages respond to *C. glabrata* infection through Syk activation and Syk-mediated release of IL-1β (Fig. 11). Second, CgYapsins impede the Syk-dependent IL-1β production/secretion (Fig. 11).

The IL-1β signaling pathway is a key component of the innate immune system that regulates the expression of hundreds of genes in a context-dependent fashion (39, 40). The NLRP3 inflammasome, which plays a crucial role in host defense against *C. albicans* through regulation of IL-1β production, is an oligomeric complex comprising NLRP3 (nucleotide-binding domain leucine-rich repeat containing receptor family member, pyrin domain–containing 3), adaptor protein ASC (apoptosis-associated speck-like protein containing CARD (caspase activation and recruitment domain), and procaspase-1 (35, 41, 42). The procaspase-1 is cleaved into an active inflammatory cysteine protease caspase-1 following assembly of the inflammasome complex (40). IL-1β secretion, upon *C. albicans*–mediated NLRP3 inflammasome activation in peripheral blood mononuclear cells, involves transcriptional induction followed by caspase-1–mediated cleavage of the pro-IL-1β cytokine (42, 43). Notably, in addition to caspase-1, other proteases, including caspase-8, neutrophil-derived serine proteases, and *C. albicans* aspartyl proteases, have also been shown to process pro-IL-1β into active IL-1β (43). Recently, *C. albicans* secreted aspartyl proteases Sap2 and Sap6 caused IL-1β production through NLRP3 and caspase-1 activation in monocytes (44). In our study, we did not find higher transcript levels of IL-1β in microarray- and qPCR-based gene expression analysis of WT– and *Cgyps1–11*Δ–infected macrophages. Hence, it is possible that either higher IL-1β secretion, upon *C. glabrata* infection, is a post-transcriptional effect, or IL-1β transcriptional induction occurs very early during infection of THP-1 cells. Further, whether CgYapsins can cleave pro-IL-1β to produce mature IL-1β remains to be determined.

The C-type lectin receptors Dectin-1 and Dectin-2 on macrophages are known to act as pattern-recognition receptors for *C. albicans* through sensing of β-glucan and α-mannan, respectively, in the fungal cell wall (24, 45). β-Glucans have been reported to induce the transcription and secretion of IL-1β via Dectin-1/Syk signaling–dependent activation of the NRLP3 inflammasome in human macrophages (41, 46). Importantly, both Dectin-1 and Dectin-2 have recently been implicated in host resistance against systemic *C. glabrata* infections, as Dectin-1– and 2–deficient mice exhibited elevated susceptibility to *C. glabrata* infections (47, 48). The key finding of involvement of Syk-dependent IL-1β production in intracellular survival in our study is likely to be dependent on extracellular/intracellular sensing of *C. glabrata* components. In this regard, it is noteworthy that *C. glabrata* cell walls contain 50% higher mannoproteins than that of *S. cerevisiae* and *C. albicans*, although β-glucan content in the *C. glabrata* cell wall is lower (23). It has also been postulated that reduced β-glucan content may help *C. glabrata* evade detection by host innate immune cells (7, 23). Although the involvement of Syk signaling and NLRP3 inflammasome activation implicates β-glucan in recognition of *Cgyps1–11*Δ cells, additional work is required to delineate the IL-1β signaling and other macrophage signaling pathways that are involved in recognition and transduction of signal evoked by *C. glabrata* cells lacking or expressing CgYapsins. Notably, infection of Raw264.7 macrophages with live *C. glabrata* cells has previously been shown to result in prolonged activation of the Syk pathway while having no effect on the NF-κB pathway (49).

Further, using the mouse model of systemic candidiasis, we demonstrate for the first time that CgYapsins are required for colonization, dissemination, and persistence of *C. glabrata* in the brain. *C. glabrata* cells have previously been harvested from brains of systemically infected mice with no change in organ burden between 2 and 7 days postinfection (11). However, the process of fungal cells trafficking to and migrating into the brain is poorly understood. *Cryptococcus neoformans* is reported to invade brain through the “Trojan horse” mechanism and start crossing the blood–brain barrier 6 h after tail vein injection (50). It has also been shown to multiply in the brain (50). Our infection kinetics data show that compared with 10^6^–10^7^ cfu in kidneys, liver, and spleen 1 dpi, mouse brains displayed only 10^4^ *C. glabrata* cfu. Furthermore, contrary to the decline in fungal burden in other organs, brain yeast cfu exhibited an increase at days 3 and 5 postinfection compared with 1 dpi. These results are consistent with findings of a previous study, wherein a small increase in brain cfu was observed from day 1 to day 3 during systemic *C. glabrata* infection of the immunocompetent mice (51). Altogether, these data do raise a possibility of modest multiplication of *C. glabrata* exclusively in the brain. Intriguingly, fungal migration/colonization and replication in the brain during early stages of systemic infection appear to be dependent on the presence of 11 CgYapsins, as the *Cgyps1–11*Δ mutant failed to reach, multiply, and persist in the brain in substantial numbers. Of note, CgYapsins have previously been implicated in colonization of *D. melanogaster* (17). How CgYapsins contribute to crossing the blood–brain barrier and modulate the immune cell response will be the focus of future investigation.

Last, we also uncover for the first time a role for CgYps2 *in vivo* and CgYps4, -5, -8, and -10 in intracellular survival *in vitro*, as the mutant lacking *CgYPS2* and *CgYPS-C* genes has previously shown no discernible phenotype (9). Although functional redundancy, due to probable evolution by the ancestral gene duplication, is not surprising in the *CgYPS* gene family, it certainly has made it difficult to ascertain the role of individual CgYapsin proteins in cell physiology. We have tried to address this by conducting studies in parallel with strains overexpressing either an individual *CgYPS* gene or lacking single, double, and multiple *CgYPS* genes. Our data reveal for the first time that CgYps2 and CgYps-C are just as important for survival in kidneys as CgYps1 and CgYps7. Additionally, the *Cgyps1*Δ*2*Δ mutant, which largely phenocopies the *Cgyps1*Δ*7*Δ and *Cgyps1*Δ*C*Δ mutants, has a phenotype more severe than that observed in the single *Cgyps1*Δ mutant. These data indicate that CgYps2 has a function of its own rather than being completely redundant, as has previously been assumed (9, 16). The unexpected lack of any intracellular survival or virulence defect of the *Cgyps2*Δ*C*Δ mutant is currently being examined; it could be due to overexpression or stabilization of CgYps1 and/or CgYps7 proteins.

Overall, our data demonstrate that lack of CgYapsins augments the inflammatory response of macrophages during infection in an IL-1β–dependent manner, thereby underscoring the regulatory role of CgYapsins in the host innate immune response (Fig. 11). Our findings also imply that regulated IL-1β cytokine production, during colonization and dissemination of *C. glabrata*, may promote recruitment of phagocytic cells that allow yeast cells to persist in the immunocompetent mice.

## Experimental procedures

### Strains, media, and growth conditions

*C. glabrata* WT and mutant strains, derivatives of the BG2 strain, were routinely grown in the rich YPD medium at 30 °C. Yeast strains carrying plasmids were cultured either in the CAA or synthetically defined YNB medium. Bacterial strains and strains carrying plasmids were grown in the LB and LB medium containing ampicillin, respectively, at 37 °C. pH of the YNB and YPD medium was adjusted with the HEPES buffer and HCl/NaOH. Logarithmic-phase cells were obtained by incubating overnight-grown cultures in fresh medium for 4 h. Strains and plasmids used in this study are listed in Tables 2 and 3.

**Table 2:** **Strains used in the study**

Yeast strain	Genotype	Reference
YRK92	*URA3 Cgyps2*Δ*::hph*	Ref. 9
YRK94	*URA3 CgypsC*Δ*::hph*	Ref. 9
YRK96	*URA3 Cgyps1*Δ*2*Δ*C*Δ*::hph*	Ref. 9
YRK97	*URA3 Cgyps7*Δ*2*Δ*C*Δ*::hph*	Ref. 9
YRK103	*ura3*Δ*::Tn903 G418R Cgyps1–11*Δ*::hph HygR*	Ref. 9
YRK126	*ura3*Δ*::Tn903 G418R Cgyps1*Δ*::hph HygR/pRK935*	Ref. 9
YRK129	*ura3*Δ*::Tn903 G418R Cgyps1–11*Δ*::hph HygR/pRK935*	Ref. 16
YRK131	*ura3*Δ*::Tn903 G418R Cgyps1–11*Δ*::hph HygR/pRK936*	Ref. 16
YRK228	*ura3*Δ*::Tn903 G418R Cgyps1*Δ*::hph HygR*	Ref. 9
YRK991	*ura3*Δ*::Tn903 G418R (BG14)*	Ref. 53
YRK1001	*URA3* (BG462)	Ref. 54
YRK1002	*URA3 Cgyps1*Δ*::hph*	Ref. 9
YRK1003	*URA3 Cgyps7*Δ*::hph*	Ref. 9
YRK1004	*URA3 Cgyps1*Δ*7*Δ*::hph*	Ref. 9
YRK1005	*URA3 Cgyps2*Δ*C*Δ*::hph*	Ref. 9
YRK1006	*URA3 Cgyps1–11*Δ*::hph*	Ref. 9
YRK1158	*ura3*Δ*::Tn903 G418R/pRK1042*	This study
YRK1162	*ura3*Δ*::Tn903 G418R Cgyps1*Δ*::hph HygR/pRK1042*	This study
YRK1165	*ura3*Δ*::Tn903 G418R Cgyps1*Δ*::hph HygR/pRK74*	Ref. 16
YRK1203	*URA3 Cgyps1*Δ*2*Δ*::hph*	Ref. 9
YRK1204	*URA3 Cgyps7*Δ*2*Δ*::hph*	Ref. 9
YRK1211	*ura3*Δ*::Tn903 G418R Cgyps1–11*Δ*::hph HygR/pRK1042*	This study
YRK1239	*URA3 Cgyps1*Δ*C*Δ*::hph*	Ref. 9
YRK1240	*URA3 Cgyps7*Δ*C*Δ*::hph*	Ref. 9
YRK1258	*ura3*Δ*::Tn903 G418R Cgyps1–11*Δ*::hph HygR/pRK74*	This study
YRK1264	*ura3*Δ*::Tn903 G418R/pRK1046*	This study
YRK1265	*ura3*Δ*::Tn903 G418R Cgyps1*Δ*::hph HygR/pRK1046*	This study
YRK1269	*ura3*Δ*::Tn903 G418R Cgyps1–11*Δ*::hph HygR/pRK1046*	This study
YRK1349	*ura3*Δ*::Tn903 G418R/pRK995*	This study
YRK2207	*ura3*Δ*::Tn903 G418R Cgyps1–11*Δ*::hph HygR/pRK1370*	This study
YRK2208	*ura3*Δ*::Tn903 G418R Cgyps1–11*Δ*::hph HygR/pRK1372*	This study
YRK2209	*ura3*Δ*::Tn903 G418R Cgyps1–11*Δ*::hph HygR/pRK1388*	This study
YRK2210	*ura3*Δ*::Tn903 G418R Cgyps1–11*Δ*::hph HygR/pRK1374*	This study
YRK2211	*ura3*Δ*::Tn903 G418R Cgyps1–11*Δ*::hph HygR/pRK1376*	This study
YRK2212	*ura3*Δ*::Tn903 G418R Cgyps1–11*Δ*::hph HygR/pRK1407*	This study
YRK2213	*ura3*Δ*::Tn903 G418R Cgyps1–11*Δ*::hph HygR/pRK1418*	This study
YRK2214	*ura3*Δ*::Tn903 G418R Cgyps1–11*Δ*::hph HygR/pRK1378*	This study
YRK2215	*ura3*Δ*::Tn903 G418R Cgyps1–11*Δ*::hph HygR/pRK1380*	This study
YRK2216	*ura3*Δ*::Tn903 G418R Cgyps1–11*Δ*::hph HygR/pRK1382*	This study
YRK2217	*ura3*Δ*::Tn903 G418R Cgyps1–11*Δ*::hph HygR/pRK1384*	This study
YRK2218	*ura3*Δ*::Tn903 G418R Cgyps1–11*Δ*::hph HygR/pRK995*	This study

**Table 3:** **Plasmids used in the study**

Plasmid	Description	Reference
pRK74	A CEN-ARS plasmid (pGRB2.2) of *C. glabrata* carrying *S. cerevisiae URA3* as a selection marker. MCS sites are flanked by *S. cerevisiae PGK1* promoter at one end and by 3′-UTR of *HIS3* at the other end	Ref. 55
pRK935	*CgYPS1 ORF* cloned in the MCS of pRK74	Ref. 9
pRK936	*CgYPS7 ORF* cloned in the MCS of pRK74	Ref. 18
pRK995	A CEN-ARS plasmid (Addgene ID 45319) of *C. glabrata* carrying *S. cerevisiae URA3* as a selection marker. MCS sites are flanked by *S. cerevisiae* promoter *HHT2* at one end and by 3′-UTR of *HIS3* at the other end	Ref. 56
pRK1042	*CgYPS1*^D378A^ cloned in the MCS of pRK74	This study
pRK1046	*CgYPS1*^D91A^ in the MCS of pRK74	This study
pRK1370	*CgYPS1* cloned in the MCS of pRK995	This study
pRK1372	*CgYPS2* cloned in the MCS of pRK995	This study
pRK1374	*CgYPS4* cloned in the MCS of pRK995	This study
pRK1376	*CgYPS5* cloned in the MCS of pRK995	This study
pRK1378	*CgYPS8* cloned in the MCS of pRK995	This study
pRK1380	*CgYPS9* cloned in the MCS of pRK995	This study
pRK1382	*CgYPS10* cloned in the MCS of pRK995	This study
pRK1384	*CgYPS11* cloned in the MCS of pRK995	This study
pRK1388	*CgYPS3* cloned in the MCS of pRK995	This study
pRK1407	*CgYPS6* cloned in the MCS of pRK995	This study
pRK1418	*CgYPS7* cloned in the MCS of pRK995	This study

### RNA-Seq analysis

For the RNA-Seq experiment, two biological replicate RNA samples were prepared from YPD-grown log-phase cells of each strain (WT and *Cgyps1–11*Δ) using the acid-phenol method, and RNA samples were sent to the Genotypic Technology's Genomic Facility (Bangalore, India). The IlluminaTruSeq RNA library protocol described in the “TruSeq RNA Sample Preparation Guide” (Part 15008136, Revision A, November 2010) was used for library preparation. Briefly, poly(A)-based mRNA purification was done on 1 μg of total RNA, and purified mRNA was fragmented for 2 min at 94 °C in the presence of divalent cations. The Superscript III reverse transcriptase and random hexamers were used for reverse transcription of RNA, and second-strand cDNA was synthesized in the presence of DNA polymerase I and RNase H. Following Illumina Adapter ligation to cDNA molecules, the library was amplified using eight cycles of PCR for enrichment of adapter-ligated fragments, and 76-base pair paired-end reads were produced with the Illumina NextSeq500. Using the *Candida* genome database as the reference genome (http://www.candidagenome.org),^3^ differentially expressed genes (>1.5-fold change and a false discovery rate–adjusted *p* value of ≤0.05) were identified from raw reads with the help of the CUFFLINKS program. The raw RNA-Seq data sets are available in the NCBI-SRA database (submission, SUB1954295; SRA, SRP090201; BioProject, PRJNA343588).

### THP-1 macrophage infection

THP-1 cells were treated with 16 nm PMA for 12 h, followed by a 12-h recovery period. Differentiated THP-1 macrophages, seeded at a density of 1 million per well of a 24-well plate, were infected to a multiplicity of infection (MOI) of 10:1 with YPD-grown *C. glabrata* cells (10^5^ cells) for all intracellular survival/replication measurement assays. After a 2-h infection, macrophages were washed three times with prewarmed sterile PBS to remove extracellular *C. glabrata* cells. Macrophages were lysed in water, and appropriate lysate dilutions were plated on YPD medium to determine the number of intracellular yeast cfu. For mixed infection assays, a cell suspension of equal numbers of WT and *Cgyps1–11*Δ cells was prepared in PBS, and 50 μl of this mixed culture (10^5^ yeast cells) was infected to THP-1 macrophages to obtain an MOI of 10:1. Total number of intracellular yeast cells at 2 and 24 h postinfection was determined by plating appropriate macrophage lysate dilutions on YPD and YPD medium containing hygromycin (500 μg/ml). The *CgYPS1* gene has been replaced with the *hph* gene in the *Cgyps1–11*Δ mutant (9), and, thus, the mutant displays resistance to hygromycin. To calculate the number of intracellular WT cells, cfu counts obtained on YPD plus hygromycin plates were subtracted from those recovered on YPD plates. To obtain dead WT and *Cgyps1–11*Δ cells, cells were heat-killed via incubation at 95 °C for 20 min. For R406 treatment, PMA-differentiated THP-1 cells were incubated with different concentrations of R406 (catalog no. HY-12067, MedChem Express) 2 h before infection with *C. glabrata* cells, and infection was continued for 24 h in the presence of R406. For MCC950 treatment, PMA-treated THP-1 macrophages were incubated with 15 μm MCC950 (catalog no. 5.38120.001, Calbiochem) 2 h before *C. glabrata* infection, and infection was performed for 24 h in the presence of MCC950.

### Human monocyte-derived macrophage infection

Human peripheral blood mononuclear cells were isolated from blood donated by healthy volunteers, using the polymorphprep^TM^ (Axis-shield, catalog no. 1114683) density gradient medium according to the manufacturer's instructions. 1 × 10^6^ peripheral blood mononuclear cells in the RPMI 1640 medium containing 1% heat-inactivated fetal bovine serum were seeded in each well of a 24-well plate. After removing nonadherent cells at 2 h, the RPMI 1640 medium containing 10% heat-inactivated fetal bovine serum and macrophage colony–stimulating factor (5 ng/ml) was added to facilitate monocyte differentiation into M2-type macrophages. After a 7-day incubation with a medium change at day 3, differentiated macrophages were infected with WT and *Cgyps1–11*Δ cells at an MOI of 10:1 following the protocol used for THP-1 cells.

### Microarray analysis

PMA-treated THP-1 cells were left uninfected or were infected either with WT or *Cgyps1–11*Δ cells at an MOI of 1:2 in a 100-mm tissue culture dish. After 2 h of infection, THP-1 cells were washed with PBS to remove noninternalized yeast cells and incubated further for 4 h followed by lysis in the TRIzol reagent (Life Technologies). RNA was extracted from two biological replicates of uninfected, WT-infected, and *Cgyps1–11*Δ–infected THP-1 cells using the TRIzol reagent as per the manufacturer's instructions. RNA was stored at −80 °C, and frozen RNA samples were sent to the Genotypic Technology's Genomic Facility (Bangalore, India). The Agilent's human GXP_8X60k array, consisting of 60-mer oligonucleotide probes, was used for hybridization of RNA. Each probe was labeled with a single color, and data were extracted using Agilent Feature Extraction software. Data were normalized using Gene Spring software. Genes were considered to be differentially expressed if -fold change in expression was ≥1.5-fold with a *p* value threshold of 0.05. Raw data sets are available at the Gene Expression Omnibus repository (accession number GSE86176).

### Immunoblotting

For Western blot analysis, PMA-differentiated THP-1 macrophages were either left uninfected or infected with WT and *Cgyps1–11*Δ cells at 1:1 MOI for 4 h and collected by scraping plate wells. Macrophages were lysed in NETN lysis buffer (250 mm NaCl, 5 mm EDTA (pH 8.0), 50 mm Tris-HCl (pH 8.0), and 0.5% Nonidet P-40) containing 1× Roche protease inhibitor and 1× phosphatase inhibitor mixture for 30 min on ice and centrifuged at 13,000 rpm for 10 min. Cell lysates containing 120 μg of protein were resolved on 10% SDS-PAGE. Anti-phospho-Syk (catalog no. 2710; CST), anti-Syk (catalog no. 2712; CST), and anti-GAPDH (ab9485, Abcam) antibodies were used to detect phosphorylated Syk, total Syk, and GAPDH, respectively.

### Site-directed mutagenesis

Using the *S. cerevisiae* Yps1 and *C. albicans* Sap9 enzymes as template, the catalytic motif of CgYps1 (602 amino acids) was determined through *in silico* analysis, and conserved aspartate residues at positions 91 and 378 were identified. These predicted catalytic residues were replaced with alanine residues through Phusion DNA polymerase-mediated PCR amplification. For this amplification, DNA of the plasmid pRK935 containing *CgYPS1* cloned in SpeI and XmaI sites was used as a template along with mutagenic primers, followed by ligation of overnight DpnI-digested PCR products. The presence of *CgYPS1*^D91A^ (*CgYPS1*^A272C^) and *CgYPS1*^D378A^ (*CgYPS1*^A1133C,C1134T^) mutations in recovered plasmids was confirmed by sequencing..

### Chitin, β-glucan, and mannan estimation

Log-phase *C. glabrata* cells were collected, washed twice with PBS, and suspended in PBS. Cells equivalent to an *A*_600_ of 2.0 were labeled for 15 min at room temperature with calcofluor white (2.5 μl, 10 mg/ml stock) for chitin estimation, with aniline blue (50 μl, 250 mg/ml stock) for β-glucan measurement, or with FITC-labeled concanavalin A (1 μl, 1 mg/ml stock) for mannan quantification and washed with PBS. Fluorescence of 50,000 labeled cells was measured using flow cytometry (BD FACS ARIA III; excitation and emission at 355 and 433 nm, respectively) and analyzed with FlowJo software. For each sample, fluorescence mean intensity of unstained cells was subtracted from that of stained cells, and the mean intensity ratio, indicating the mutant/WT fluorescence intensity values, was calculated and plotted.

### Cytokine measurement

Estimation of cytokines, secreted by THP-1 macrophages, upon infection with *C. glabrata* cells, was first performed using the Multi-Analyte ELISArray kit (MEH-004A, Qiagen), which analyzes a panel of 12 cytokines, IL-1α, IL-1β, IL-2, IL-4, IL-6, IL-8, IL-10, IL-12, IL-17A, IFN-ɑ, TNFα, and GM-CSF. For the assay, THP-1 macrophages were either left uninfected or infected with WT/*Cgyps1–11*Δ cells at an MOI of 1:1. Extracellular yeast cells were removed at 2 h postinfection, followed by incubation of infected and uninfected macrophages in fresh prewarmed RPMI medium. At 24 h postinfection, medium was collected and centrifuged at 1000 × *g* for 10 min to remove any cell debris, and the supernatant (100 μl) was used for cytokine measurement. For determination of IL-1β levels in culture supernatants of R406-treated and MCC950-treated *C. glabrata*–infected macrophages, the human IL-1β ELISA Set II kit (BD Biosciences catalog no. 557953) was used as per the manufacturer's instructions.

IL-1β cytokine measurement in mouse organ homogenates, following the protocol of Amsen *et al.* (52), was done using the mouse IL-1β/IL-1F2 DuoSet ELISA kit (R&D Systems, catalog no. DY401-05). Mouse kidneys, liver, spleen, and brain were homogenized in 1 ml of PBS. 500 μl of homogenate was lysed with 500 μl of tissue lysis buffer (10 mm Tris-Cl (pH 8.0), 150 mm NaCl, 1% NP-40, 10% glycerol, 5 mm EDTA, and protease inhibitor mixture) for 60 min at 4 °C, followed by sonication. Lysates were centrifuged at 14,000 rpm for 15 min at 4 °C, and cytokine levels were measured in the supernatant (100 μl) following the manufacturer's instructions.

### Mice infection assay

Mice infection experiments were performed at the CDFD Animal Facility, VIMTA Labs Ltd. (Hyderabad, India) in compliance with guidelines of the Committee for the Purpose of Control and Supervision of Experiments on Animals (CPCSEA), Government of India. The protocol was approved by the Institutional Animal Ethics Committee of Vimta Labs Ltd. *C. glabrata* strains were grown overnight in YPD at 30 °C, washed, and suspended in PBS. 100 μl of PBS cell suspension (4 × 10^7^ cells) were injected into tail veins of 6–8-week-old female BALB/c mice. At 1, 3, 5, and 7 days postinfection, animals were euthanized using carbon dioxide, and four target organs (kidneys, liver, brain, and spleen) were collected. Organs were homogenized in 1 ml of PBS, and yeast fungal load was determined by plating appropriate dilutions of tissue homogenates on YPD medium containing penicillin and streptomycin antibiotics.

For histopathology studies, two uninfected control, two WT*-*infected, and two *Cgyps1–11*Δ–infected mice were sacrificed, and organs (kidneys, liver, spleen, and brain) were harvested and fixed in 3.5% *p*-formaldehyde for 24 h. Collected tissues were trimmed and processed in an automatic tissue processor (Microm spin tissue processor STP 120-3), followed by embedding in paraffin wax using the Tissue Embedding System (Microm Embedding Center EC350) at VIMTA Labs Ltd. Embedded tissues were further trimmed with the help of an automatic microtome (Microm HM355), and thin sections of 3–5 μm were cut. These sections were placed on clean prelabeled grease-free glass slides and stained either with hematoxylin and eosin in an automatic tissue stainer (Microm HMS 740 robotic stainer) or manually with PAS.

### Other methods

qPCR and the serial dilution spotting assay were performed as described previously (9, 16).

### Statistical analysis

GraphPad Prism software was used to perform statistical analysis. The nonparametric Mann–Whitney test was used to analyze fungal burden in murine organs. The two-tailed Student's *t* test was used for intergroup comparisons.

## Author contributions

M.R. and R.K. conceived and designed the study. M.R. and A.B. acquired and analyzed data. M.R., A.B., and R.K. wrote the manuscript.

## Supplementary Material

Supporting Information

## Footnotes

5 Please note that the JBC is not responsible for the long-term archiving and maintenance of this site or any other third party hosted site.

## Footnotes

4 The abbreviations used are:

BSI: bloodstream infection
GPI: glycosylphosphatidylinositol
GO: gene ontology
qPCR: quantitative real-time PCR
MAPK: mitogen-activated protein kinase
PI3K: phosphatidylinositol 3-kinase
TNF: tumor necrosis factor
IL: interleukin
IFN: interferon
GM-CSF: granulocyte-macrophage colony-stimulating factor
dpi: days postinfection
PAS: periodic acid–Schiff
MMS: methyl methane sulfonate
PMA: phorbol 12-myristate 13-acetate
MOI: multiplicity of infection
GAPDH: glyceraldehyde-3-phosphate dehydrogenase
DEG: differentially expressed gene.
