# Supplementary material for: Aspartyl proteases in Candida glabrata are required for suppression of the host innate immune response
Source: J Biol Chem. 2018 Feb 28;293(17):6410–33. doi: 10.1074/jbc.M117.813741 (PMC5925793; doi:10.1074/jbc.M117.813741)
Supplement: Supporting Information [file supp_293_17_6410__index.html]

Aspartyl proteases in Candida glabrata are required for suppression of the host innate immune response — Aspartyl proteases in Candida glabrata are required for suppression of the host innate immune response — Role of fungal yapsins in host immune response — Supporting Information 

# Aspartyl proteases in *Candida glabrata* are required for suppression of the host innate immune response

## Supporting Information

- Supplemental Tables S1-3 (.xlsx, 71 KB) - Table S1: List of differentially expressed genes in the Cgyps1-11??? mutant Table S2: List of differentially expressed genes in wild-type-infected THP-1 macrophages Table S3: List of differentially expressed genes in Cgyps1-11???-infected THP-1 macrophages
